# PRKN/parkin-mediated control of SNCA (synuclein alpha) and chaperone-mediated autophagy are defective in cellular, mice models and Parkinson disease-affected brains

**DOI:** 10.1080/15548627.2026.2669458

**Published:** 2026-05-13

**Authors:** Lígia Ramos dos Santos, Eric Duplan, Juliane Debord, Gwendoline Fremont, Julien Minniti, Inger Lauritzen, Eugenie Mutez, Coline Leghay, Marie Christine Chartier-Harlin, Frédéric Checler, Cristine Alves da Costa

**Affiliations:** a University Côte d’Azur, INSERM, CNRS, Institut de Pharmacologie Moléculaire et Cellulaire, “Laboratory of Excellence (LABEX) Distalz”, Valbonne, France; b University of Lille, INSERM, CHU Lille, UMR-S 1172-LilNCog-Lille Neuroscience and Cognition, Lille, France

**Keywords:** chaperone mediated autophagy, *GBA1* and *SNCA* gene regulation, *in vivo* studies, PRKN role in CMA, PRKN transcription factor function

## Abstract

Pathological accumulation of toxic SNCA species and loss of E3-ligase function of PRKN are two key features observed in Parkinson disease (PD). Here, we established the contribution of an E3-ligase-independent transcriptional function of PRKN in SNCA regulation. PRKN depletion decreased *SNCA* and *GBA1* (glucosylceramidase beta 1) mRNA levels and reduced CMA-driven degradation of SNCA, thereby triggering the accumulation of its phosphorylated aggregation-prone toxic species. We established that PRKN controls the CMA player LAMP2A but not HSPA8/HSC70 in isolated lysosomal fractions prepared from human neuronal and mouse fibroblastic cells. Further, we showed that PRKN-associated regulation of LAMP2 is isoform specific. We showed that PRKN-mediated control of SNCA, GBA1 and LAMP2A occurs *in vivo* and is impaired in the paraquat-treated PD mice model. We showed that the levels of phosphorylated SNCA and PRKN are correlated in sporadic PD human brain samples and that fibroblasts of patients carrying pathogenic *PRKN* mutations exhibit impaired CMA activity. Our study decrypts a new molecular mechanism linking three PD major therapeutic targets. It enriches the portfolio of transcriptional targets of PRKN and establishes PRKN as a novel CMA regulator. Further, it shows that PRKN controls both direct and indirect (GBA1-dependent) transcriptional regulation of *SNCA*. This novel molecular cascade opens potential new avenues in PD treatment.

**Abbreviations:** ChIP: chromatin immunoprecipitation; CMA: chaperone-mediated autophagy; ΔPkPr: deleted *PRKN*-RE *Snca* promoter; GBA1: glucosylceramidase beta 1; HAP1: human haploid cell; *GBA1*^*+*^: human haploid control cells; *GBA*^−^: human haploid invalidated for *GBA1* cells; HSPA8/HSC70: heat shock protein family A (Hsp70) member 8; LAMP2A: lysosomal associated membrane protein 2A; MEF: mouse embryonic fibroblast; *Prkn*^*+/+*^: MEF and mice *Prkn* control; *prkn*^*-/-*^: MEF and mice *prkn* knockout; PD: Parkinson disease; PQ: paraquat; PRKN: parkin RBR E3 ubiquitin protein ligase; *PRKN*-RE: *PRKN* responsive element; *PRKN [SC]*: PRKN control; *PRKN [KD]*: *PRKN*-depleted; SNCA: synuclein alpha; SNCA [M]: monomeric synuclein alpha; SNCA [O]: oligomeric synuclein alpha; SNCA p-S129: phosphorylated synuclein alpha, SPD: sporadic Parkinson disease; TF: transcription factor; TH: tyrosine hydroxylase; *WTPr*: wild-type *Snca* promoter.

## Introduction

Parkinson disease (PD) is a multi-causal movement disorder characterized by the degeneration of dopaminergic neurons of the *substantia nigra pars compacta*. The pathophysiology of PD is complex and likely accounted for by various cellular dysfunctions that include among others, exacerbated endoplasmic reticulum stress and altered unfolded protein response as well as proteasomal and autophagy machineries failures. Interestingly, altered autophagic defects are generally associated to the accumulation of toxic aggregated proteins. It is the case for SNCA (synuclein alpha) [1–3], a protein responsible for a major subset of autosomal dominant familial cases of PD [4]. SNCA is a natively unfolded protein, which constitutes the major filamentous component of Lewy bodies that are the main histopathological hallmarks of PD [5]. The precise function of SNCA is not yet fully established, but its ability to control synaptic transport mechanisms has been consistently described [6]. In physiological conditions, SNCA occurs as non-aggregable monomeric species, which are associated to a neuroprotective role [7–9]. However, under several pathological conditions including various types of oxidative stresses and presence of aggregation, it loses its neuroprotective function [8,10]. Thus, the understanding of the molecular mechanisms responsible for the control of synuclein alpha protein homeostasis is essential to better understand PD etiology. Recent studies documented the fact that GBA1 (glucosylceramidase beta 1), contributed to the chaperone-mediated autophagy (CMA)-linked degradation of SNCA. Of note, it was recently demonstrated that mutations of *GBA1*, which correspond to the most common risk factor to develop Parkinson disease, impair SNCA degradation [11] and thereby, likely trigger its accumulation and subsequent aggregation.

Previous data indicated that *SNCA* mRNA levels appear reduced in PD-affected brains [12–14], in apparent contradiction with the notable increase in its aggregated, toxic oligomeric/fibrillar species [15]. This paradoxical dichotomy between protein and RNA levels remained a puzzling mechanistically unsolved question. Intuitively, one could envision that the reduction of *SNCA* mRNA levels indeed reduces SNCA monomeric expression, but that putative concomitant impairment of SNCA monomers degradative mechanisms may overall account for the augmentation of aggregated species demonstrated in PD. This hypothesis implying alterations of both *SNCA* gene transcription and CMA degradation, remained to be experimentally demonstrated. Here, we unravel a molecular cascade resolving this paradox by establishing that PRKN (parkin RBR E3 ubiquitin protein ligase) is a key regulator of SNCA and of CMA.

Our data stand on our previous demonstration that besides its canonical role as an E3-ligase, PRKN, which is responsible for most of autosomal recessive Parkinson disease cases [16,17], could also act as a genuine transcription factor (TF) [17–19]. During the course of our study on PRKN TF function, we delineated a *PRKN*-responsive element (*PRKN*-RE) [18]. Interestingly, an *in silico* analysis of the promoters of *SNCA* and *GBA1*, allowed us to identify this putative functional *PRKN*-RE, suggesting that *SNCA* and *GBA1* promoters could well behave as novel PRKN transcriptional targets. Indeed, our work demonstrates that PRKN acts as a direct regulator of *SNCA* and *GBA1* transcription in cells as well as *in vivo* and controls the CMA in physiological conditions. Of importance, we establish that this control is altered in pharmacologically-induced experimental PD condition. Thus, overexpression of PRKN triggered an upregulation of SNCA neuroprotective monomers and a down-regulation of aggregated toxic SNCA by a transcriptional mechanism implying PRKN binding to *SNCA* and *GBA1* promoters. Importantly, this two-hits regulation may explain the opposite changes to SNCA protein and mRNA levels in PD. Moreover, we showed a hitherto undescribed implication of PRKN in an additional autophagy type, namely CMA. Thus, PRKN increased CMA activity by modulating LAMP2A/*LAMP2A* but not *LAMP2B, LAMP2C*, LAMP1, and HSPA8 levels and contributed to GBA1-mediated SNCA degradation by CMA. Of note, experimental PD triggered by classical paraquat treatment of mice impaired PRKN-linked control of SNCA, GBA1 and LAMP2A protein expressions. Of most importance, we established that phosphorylated SNCA and PRKN levels are negatively correlated in sporadic PD-affected brain samples and that fibroblasts of patients carrying PRKN mutations indeed showed altered CMA activity.

Overall, our study describes a novel signaling cascade linking three main PD-related therapeutic targets, namely PRKN, GBA1 and SNCA and shows that this functional interplay is severely altered in PD condition. Further, it demonstrates for the first time that PRKN is not only involved in the control of mitophagy but also in CMA. The ability of PRKN to increase CMA and to regulate the levels of monomeric and aggregated SNCA further supports its neuroprotective role in physiological conditions and suggests that the impairment of PRKN TF function could explain several well-documented PD alterations linked to SNCA and CMA defects.

## Results

### Overexpressed and endogenous PRKN control the level of SNCA in vitro and ex vivo

**Figure 1.**
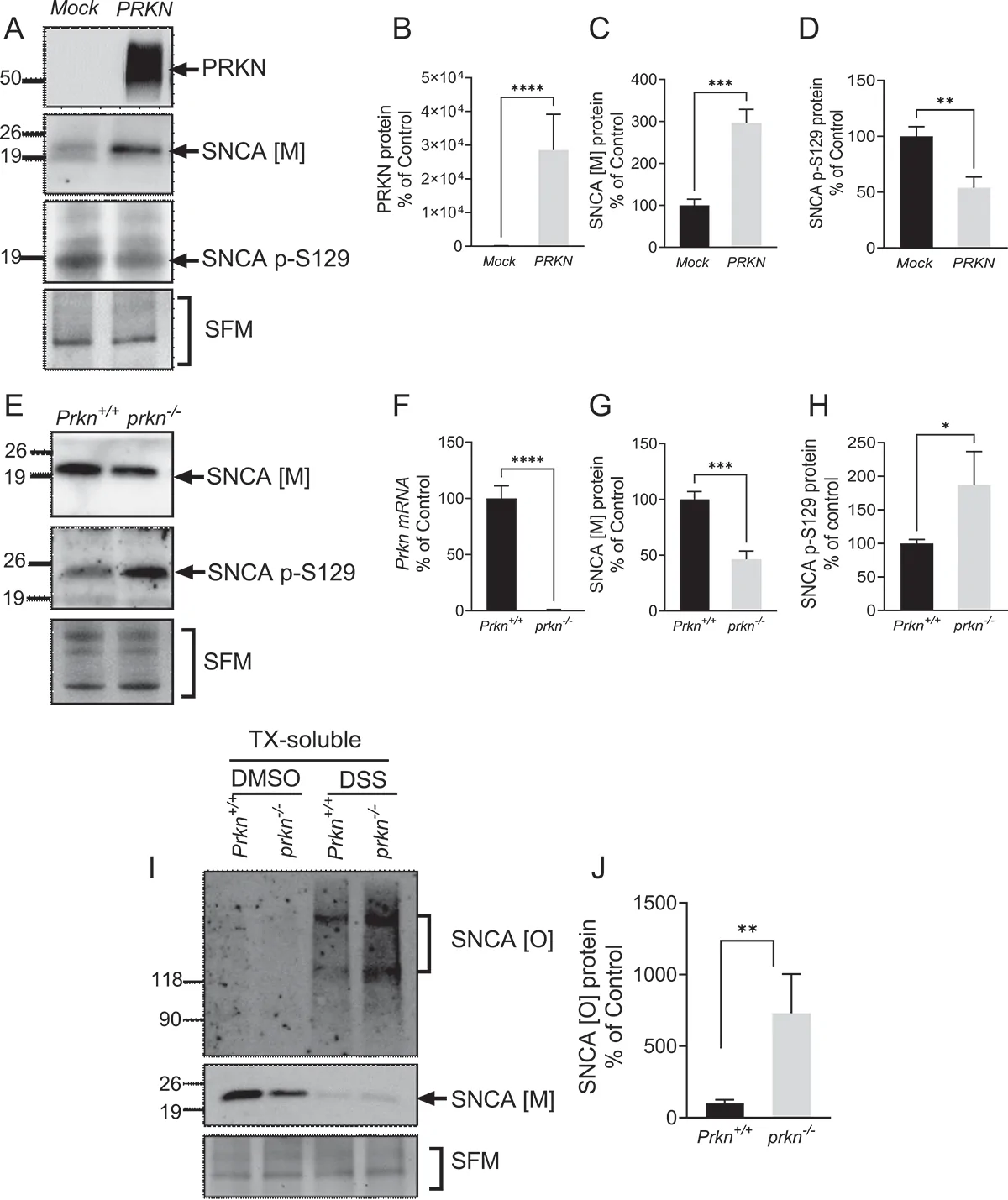
PRKN regulates monomeric, phosphorylated and oligomeric SNCA expression. SH-SY5Y cells overexpressing *PRKN* (A-D) or wild-type *Prkn*^+/+^ and *prkn*^−/−^ mouse fibroblasts (E-H) were assessed and quantified for monomeric (SNCA [M], (A, C, E and G) and phosphorylated (SNCA p-S129], (A, D, E and H) protein expression by western blot and *prkn* mRNA (F) as described in the materials and methods. (I and J) *Prkn*^+/+^ and *prkn*^−/−^ fibroblasts were assessed and quantified for oligomeric SNCA [O] as described in the methods. In all experiments, proteins loading is assessed by stain free membranes (SFM) as described in the materials and methods. All bars are expressed as percent of mock (B-D) or *Prkn*^+/+^ (F-H, J) (taken as 100) and are the means ± SEM. *p < 0,05; **p < 0.005; ***p < 0.001; ****p < 0.0001. ns, not statistically significant. Full gels are provided in data S1.

Based on the identification of a *PRKN*-RE on the *SNCA* promoter, we first aimed at examining the putative involvement of PRKN in the control of monomeric (neuroprotective) and aggregated (neurotoxic) SNCA expression in the human neuronal cell line SH-SY5Y, a dopaminergic cell line widely used as a PD-relevant model [20]. Figure 1 shows that PRKN overexpression (Figure 1(A,B), *N =* 12, Mann Whitney test, 4 independent experiments performed in triplicate) triggered a significant increase of monomeric SNCA (SNCA [M]) expression (Figure 1(A,C), *N =* 9, Mann-Whitney test, 3 independent experiments performed in triplicate) while its aggregable toxic phosphorylated (SNCA p-S129) form was decreased (Figure 1(A,D), *N =* 8, Mann-Whitney test, 4 independent experiments performed in duplicate). Conversely, full knockout of *Prkn* in primary mouse embryonic fibroblasts (see Figure 1(F), *N =* 12, Mann Whitney test, MEF, *prkn*^*-/-*^, 3 independent experiments performed in triplicate) triggered a reduction of SNCA [M] (Figure 1(E,G), *N =* 8, t-test, 4 independent experiments performed in duplicate) and an increase of SNCA p-S129 (Figure 1(E,H), *N* = 5, Mann-Whitney test, 2 independent experiments performed in duplicate/triplicate) expression. Of note Figure 1(I) confirmed the reduction of SNCA [M] in DSS-treated TX-soluble fraction prepared from *prkn*^*-/-*^ cells but also revealed a concomitant drastic enhancement of SNCA oligomeric forms (see SNCA [O] in Figure 1(I,J), *N =* 6, Mann-Whitney test, 3 independent experiments performed in duplicate). The regulation of SNCA expression by endogenous PRKN was further confirmed in *PRKN*-knocked down SH-SY5Y cells (*PRKN* [KD]), see reduction of endogenous PRKN protein expression in Figure S1A and B), in which decreased SNCA monomeric protein expression (Figure S1A and C, *N =* 8, Mann-Whitney test, 4 independent experiments performed in duplicate) was also observed.

### PRKN controls SNCA at a transcriptional level

**Figure 2.**
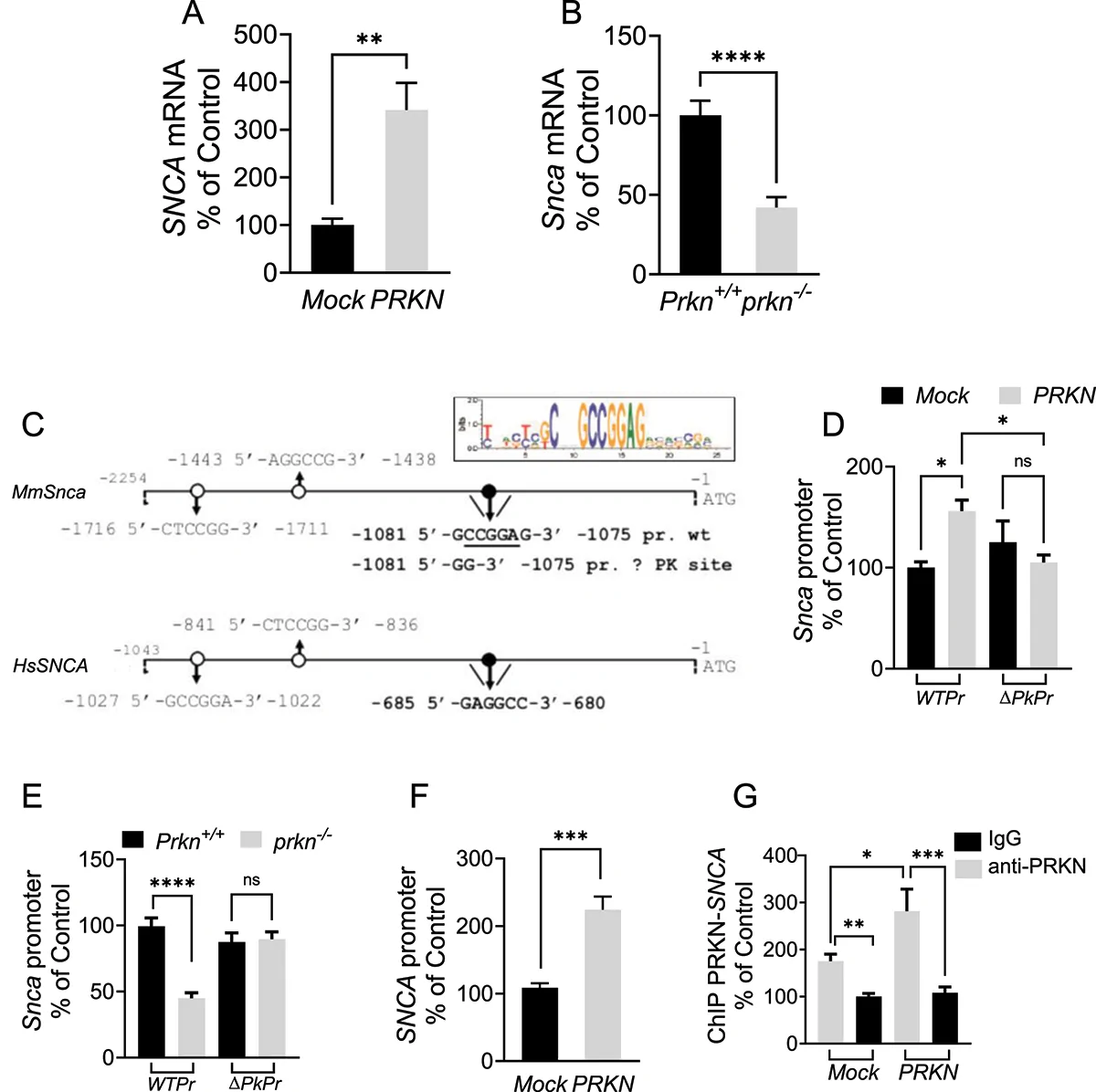
PRKN is a direct transcriptional regulator of SNCA. Human (A) or murine (B) *SNCA/Snca* mRNA expressions (respectively) are measured in Mock or *PRKN*-transfected SH-SY5Y (A) and in *Prkn*^+/+^ or *prkn*^−/−^ fibroblasts (B). Values are expressed as percent of control (Mock/*Prkn^+/+^*) cells (taken as 100) and are the means ± SEM. (C) schematic representation of murine *Snca* (*MmSnca*, upper panel) and human *SNCA* (*HsSNCA*, lower panel) promoter regions where putative *PRKN-*RE sites are indicated by empty or black circles. The *PRKN-*RE site on *Snca* indicated by black circle was deleted (*ΔPKPr*) by site direct mutagenesis as described in materials and methods. (D and E) wild-type (*WTPr*) and *ΔPkPr* promotor activities were measured in *Mock*- or *PRKN*-transfected SH-SY5Y (D) or in *Prkn*^+/+^ or *prkn^−/−^* fibroblasts (E). (F) activity of the wild-type human *SNCA* promoter encompassing the three *PRKN-*RE was measured in control (*Mock*) and *PRKN*-transfected SH-SY5Y cells. Bars in D-F are expressed as percent of controls (*Mock/WTPr* or *Prkn*^+/+^/*WTPr*) (taken as 100) and are the means ± SEM. (G) ChIP analysis of the physical interaction between endogenous (Mock) or overexpressed PRKN and *PRKN-*RE- (5’-GAGGCC-3,’ black circle in C, lower panel) containing probe was assessed by means of control (IgG, black bars) or PRKN-directed antibodies (anti-PRKN, gray bars) as described in materials and methods. Bars represent the means ± SEM. * *p* ≤ 0.05; **p ≤ 0.01; ***p ≤ 0.001; ****p ≤ 0.0001; ns: not statistically significant.

To assess whether the transcriptional function of PRKN could account for PRKN-linked SNCA regulation, we examined the effect of PRKN overexpression and depletion on human/mouse *SNCA/Snca* mRNA levels. In SH-SY5Y cells, PRKN overexpression leaded to an about 3-fold increase of *SNCA* mRNA levels (Figure 2(A), *N =* 6, Mann Whitney test, 3 independent experiments performed in duplicate) while *prkn/PRKN* gene knockout/knockdown in MEF primary cells (Figure 2(B), *N =* 16, t-test, 4 independent experiments performed in quadruplicate) and SH-SY5Y (Figure S1D, *N* = 15 Mann-Whitney test, 5 independent experiments performed in triplicate) cells, respectively, drastically reduced *SNCA/Snca* mRNA levels. These data suggest that both human and murine *SNCA/Snca* promoters are putative PRKN direct targets in both overexpression and physiological conditions. Supporting this view, we identified three putative *PRKN*-responsive elements (*PRKN*-RE) on the mouse *Snca* promoter sequence (*MmSnca*, Figure 2(C), upper panel, see sites 1, 2 and 3). We examined the influence of the deletion of the most reliable (see logo obtained by GENOMATIX program) *PRKN*-RE identified *in silico* (site 1, black circle, −1081/-1075, construct referred to as *ΔPkPr*) on the ability of PRKN to regulate the *Snca* promoter. As expected, PRKN overexpression led to a significant increase of the murine wild-type *Snca* promoter activity (*WTPr*) while *ΔPkPr* promoter activity was fully abolished (Figure 2(D), *N =* 12, One-way ANOVA, Tukey’s multiple comparison test, 4 independent experiments performed in triplicate). A similar conclusion applies to primary MEF cells where *prkn* gene knockout reduced *WTPr* but not *ΔPkPr* promoter activity (Figure 2(E), *N =* 16, One-way ANOVA, Sidak’s multiple comparison test, 4 independent experiments performed in quadruplicate). The close *in silico* analysis of the human and mouse *SNCA/Snca* promoters showed that they present a good homology (49%) and that the human *SNCA* promoter also harbors three *PRKN*-REs (*HsSNCA*, Figure 2(C), lower panel). Thus, we have cloned the human *SNCA* promoter and examined its regulation by PRKN. We show that, like the murine counterpart, the human *SNCA* promoter is significantly upregulated upon PRKN overexpression in SH-SY5Y cells (Figure 2(F), *N =* 12, t-test, 4 independent experiments performed in triplicate). The responsiveness of murine and human *Snca/SNCA* promoters were also observed in PRKN knockdown SH-SY5Y cells (Figure S1E, *N =* 6–12, t-test, 2 to 4 independent experiments performed in triplicate). Again, we confirmed that *ΔPkPr* activity was no longer regulated by PRKN in *PRKN [KD]* cells (Figure S1F, *N =* 12, One-way ANOVA, Sidak’s multiple comparison test, 4 independent experiments performed in triplicate).

To establish whether PRKN controls *SNCA* transcription directly, we have evaluated the ability of PRKN to physically interact with the human *SNCA* promoter. Thus, we have designed chromatin immunoprecipitation (ChIP) primers targeting the sites 1–3 *in silico* mapped *PRKN*-REs and we have identified site 3 (Figure 2(G), *N =* 6, one-way ANOVA, Sidak’s multiple comparison test, 3 independent experiments performed in duplicate) but not sites 1 (Figure S2A, *N=*6, one-way ANOVA, Sidak’s multiple comparison test, 3 independent experiments performed in duplicate) and 2 (Figure S2B, *N =* 6, one-way ANOVA, Sidak’s multiple comparison test, 3 independent experiments performed in duplicate) as the one allowing direct physical interaction with the *human SNCA* promoter in PRKN-expressing conditions. Overall, the above-described data unravel the functional relevance of the *PRKN*-RE and thus demonstrate that the *SNCA* promoter behaves as a direct transcriptional target of endogenous PRKN.

### PRKN controls GBA1 transcription

**Figure 3.**
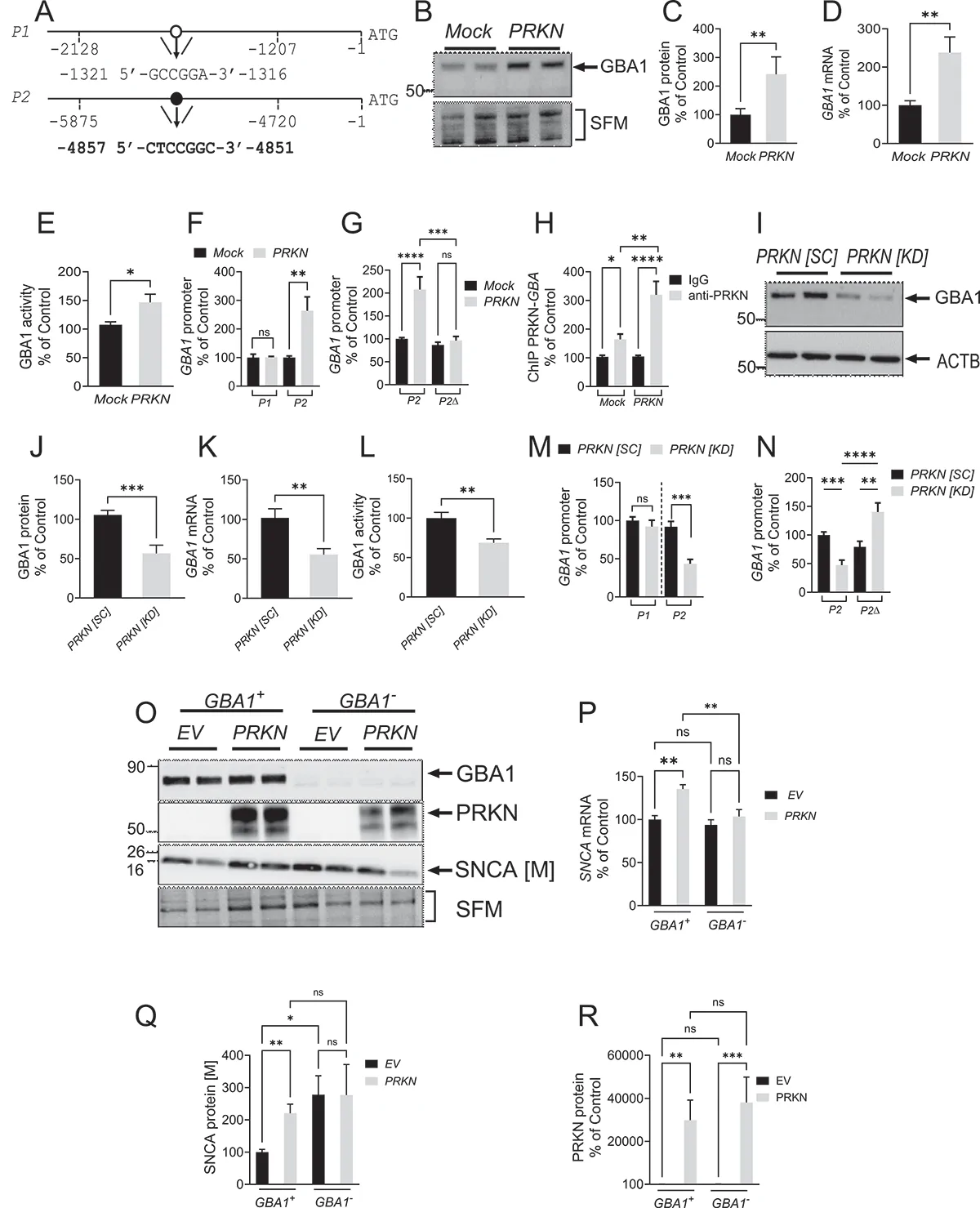
Direct transcriptional regulation of GBA1 and indirect GBA-dependent control of *SNCA* by PRKN. (A) schematic representation of the human promoters (*P1* and *P2*) of *GBA1* encompassing similar *PRKN-*RE sites. *Mock* or *PRKN*-transfected SH-SY5Y cells (B-E) or stably expressing scramble (*PRKN [sc] or PRKN*-directed *shRNA* (*PRKN [KD*], I-L) were assessed for GBA1 protein expression (b, C, I and J). Proteins loading was assessed either by stain free membranes (sfm) or ACTB (actin beta). where indicated. Conduritol-β-epoxide-sensitive GBA1 enzymatic activity (E and L) and *GBA1* mRNA levels (D and K) were measured as described in materials and methods. Bars in C-E and J-L are expressed as percent of control *Mock* or *PRKN [sc]* cells (taken as 100) and the means ± SEM. *P1* and *P2* activities were measured in *PRKN*-transfected (F) and *PRKN [KD*] cells (M). Bars are expressed as percent of *P1*/Mock or *P1/PRKN [sc]* conditions (taken as 100) and are the means ± SEM. The *PRKN*-RE site on *P2* was deleted (*P2Δ*) by site directed mutagenesis then *GBA1* promoter activity was measured in Mock- and *PRKN*- transfected cells (G) and in *PRKN [sc]* and *PRKN [KD*] cells (N). Values are expressed as percent of controls *Mock/P2* (G) or *PRKN [sc] /P2* (N) taken as 100 and are the means ± SEM. Physical interaction between endogenous (*Mock* condition) or overexpressed PRKN and *GBA1 P2* promoter was assessed (H) by means of control (IgG, black bars) or PRKN-directed antibodies (gray bars) as described in materials and methods. *p ≤ 0.05; ** *p* ≤ 0.01; ***p ≤ 0.001; ***p ≤ 0.001; ns: not statistically significant. (O-R) *GBA1^+^* and *GBA1*^−^ HAP1 cells were transiently transfected with empty vector (EV) or *PRKN* then analyzed for GBA1 (O), PRKN (O and R), SNCA [M] (O and Q) protein expressions and *SNCA* mRNA (P). Values are expressed as percent of controls (*EV/GBA1^+^*) taken as 100 and are the means ± SEM. Full gels are provided in data S2.

GBA1 is involved in CMA-mediated SNCA degradation and PD-related GBA1 mutations have been associated with oligomeric SNCA accumulation in affected human brains [21]. Thus, the examination of the possible impact of PRKN on *GBA1* transcriptional regulation remains of utmost importance for understanding the mechanisms underlying the accumulation of aggregated SNCA and the well-documented decrease of its mRNA levels in PD. Our *in silico* study identified the presence of several putative *PRKN-*REs in the human promoters *1* and *2* of *GBA1* (*P1* and *P2* Figure 3(A)). This led us to hypothesize that reduced PRKN activity in PD could impact/lower GBA1 protein levels and activity, and by consequence, exacerbate the accumulation of neurotoxic aggregated forms of SNCA. Supporting this hypothesis, Figure 3(B–E) shows that PRKN overexpression increased GBA1 protein expression (Figure 3(B,C) *N =* 12, Mann-Whitney test, 4 independent experiments performed in triplicate), mRNA levels (Figure 3(D), *N =* 6, Mann-Whitney test, 3 independent experiments performed in duplicate) and enzymatic activity (Figure 3(E), *N =* 9, t-test, 3 independent experiments performed in triplicate) in SH-SY5Y. Next, we cloned the two human *GBA1* promoters and evaluated the effect of PRKN overexpression on their regulations in SH-SY5Y cells. Figure 3(F) (*N* = 6–9, Mann-Whitney test, 3 independent experiments performed in duplicate/triplicate) showed that only the *P2* promoter activity was drastically increased by PRKN overexpression, indicating that the putative *PRKN*-RE of *P1* promoter was not functional in our experimental setting. Consistent with this conclusion, the deletion of the *PRKN*-RE of the *P2* promoter completely abolishes PRKN-mediated regulation of *P2* activity in SH-SY5Y cells (Figure 3(G), *N =* 12, one-way ANOVA, Sidak’s multiple comparison test, 4 independent experiments performed in triplicate). Finally, we analyzed PRKN ability to interact physically with the functionally validated −4857/-4851 region of the *P2* promoter by ChIP assay. Figure 3(H) shows that both endogenous and overexpressed PRKN interacted with the *P2* promoter region containing the *PRKN*-RE (−4857/-4851) (*N =* 8, Kruskal Wallis test, Dunn’s multiple comparison test, 4 independent experiments performed in duplicate), thus demonstrating that *GBA1* behaves as a genuine PRKN transcriptional target. Similar observations stand in SH-SY5Y cells where *PRKN* gene was knocked-down (*PRKN [KD]*) by shRNA approach. Thus, PRKN reduction triggers a significant decrease of GBA1 protein (Figure 3(I,J), *N =* 16, t-test, 4 independent experiments performed in quadruplicate), mRNA (Figure 3(K), *N =* 10, t-test, 2 independent experiments performed in quintuplicate) expression and activity (Figure 3(L), *N =* 12, t-test, 4 independent experiments performed in triplicate). Consistent with the above overexpression data, only *P2* promoter activity was lowered by the reduction of endogenous PRKN in SH-SY5Y cells (Figure 3(M), *N =* 9, Mann-Whitney test, 3 independent experiments performed in triplicate). Consistent with this conclusion, the deletion of the PRKN-RE of the *P2* promoter completely abolishes PRKN-mediated regulation of P2 activity in SH-SY5Y cells (Figure 3(N), *N=*12, one-way ANOVA, Sidak’s multiple comparison test, 4 independent experiments performed in triplicate). Further supporting the above conclusions, Figure S3 shows that a full knockout of the *Prkn* gene in primary MEF cells led to a significant reduction of GBA1 protein (Figure S3A and B, *N* = 9, t-test, 3 independent experiments performed in triplicate), GBA1 activity (Figure S3C, *N =* 7, Mann-Whitney test, 2 independent experiments performed in tri- and quadruplicate), mRNA levels (Figure S3D, *N =* 16, t-test, 4 independent experiments performed in quadruplicate) and *P2* but not *P1 GBA1* promoter activities (Figure S3E, *N =*1 1, t-test, 3 independent experiments performed in triplicate and 1 in duplicate). Further, we confirmed that the deletion of the *PRKN*-RE in *GBA1 P2* abolished PRKN ability to control *GBA1 P2* promoter (Figure S3F, *N =* 12, one-way ANOVA, Sidak’s multiple comparison test, 4 independent experiments performed in triplicate). Overall, the above set of data clearly identify a functional *PRKN*-RE responsible for direct PRKN-mediated transcriptional regulation of *GBA1*.

### PRKN-mediated control of SNCA is GBA1-dependent

Overall, our data suggest a direct transcriptional control of *SNCA* and *GBA1* by PRKN, but do not preclude an additional functional cascade linking indirectly PRKN to SNCA via GBA1. We took advantage of available human haploid 1 (HAP1) control (*GBA1*^+^) and knockout (*GBA1*^−^) cells to examine this hypothesis. Transient overexpression of PRKN in *GBA1*^+^ cells (see Figure 3(O,P)) induced an increase of SNCA [M] (Figure 3(O,Q), *N =* 15, Kruskal-Wallis test, Dunn’s multiple comparison test, 5 independent experiments performed in triplicate). As was expected, in absence of *PRKN* cDNA transfection, *GBA1* knockout led to the expected increase of SNCA [M] (Figure 3(O,Q), *N =* 15 compare EV black bars, 5 independent experiments performed in triplicate), confirming the consistent reports on the contribution of endogenous GBA1 in the control of SNCA degradation and thereby, its protein levels. Of importance, the depletion of endogenous *GBA1* gene fully abolished PRKN-mediated control of SNCA [M] protein expression (Figure 3(Q), see PRKN expressions in Figure 3(R)), indicating that PRKN-dependent modulation of SNCA is GBA1-dependent in HAP1 cells. Of note, *GBA1* deletion impaired the ability of PRKN to regulate *SNCA* mRNA (Figure 3(P), *N* = 12, one-way ANOVA, Sidak’s multiple comparison test, 4 independent experiments performed in triplicate). This set of data indicates that, although PRKN directly modulates *SNCA* mRNA and SNCA protein expressions in physiological conditions, the GBA1-dependent contribution of PRKN-regulated control of SNCA appears as the main mechanism responsible for the regulation of SNCA levels.

### PRKN contributes to the control of chaperone-mediated autophagy

**Figure 4.**
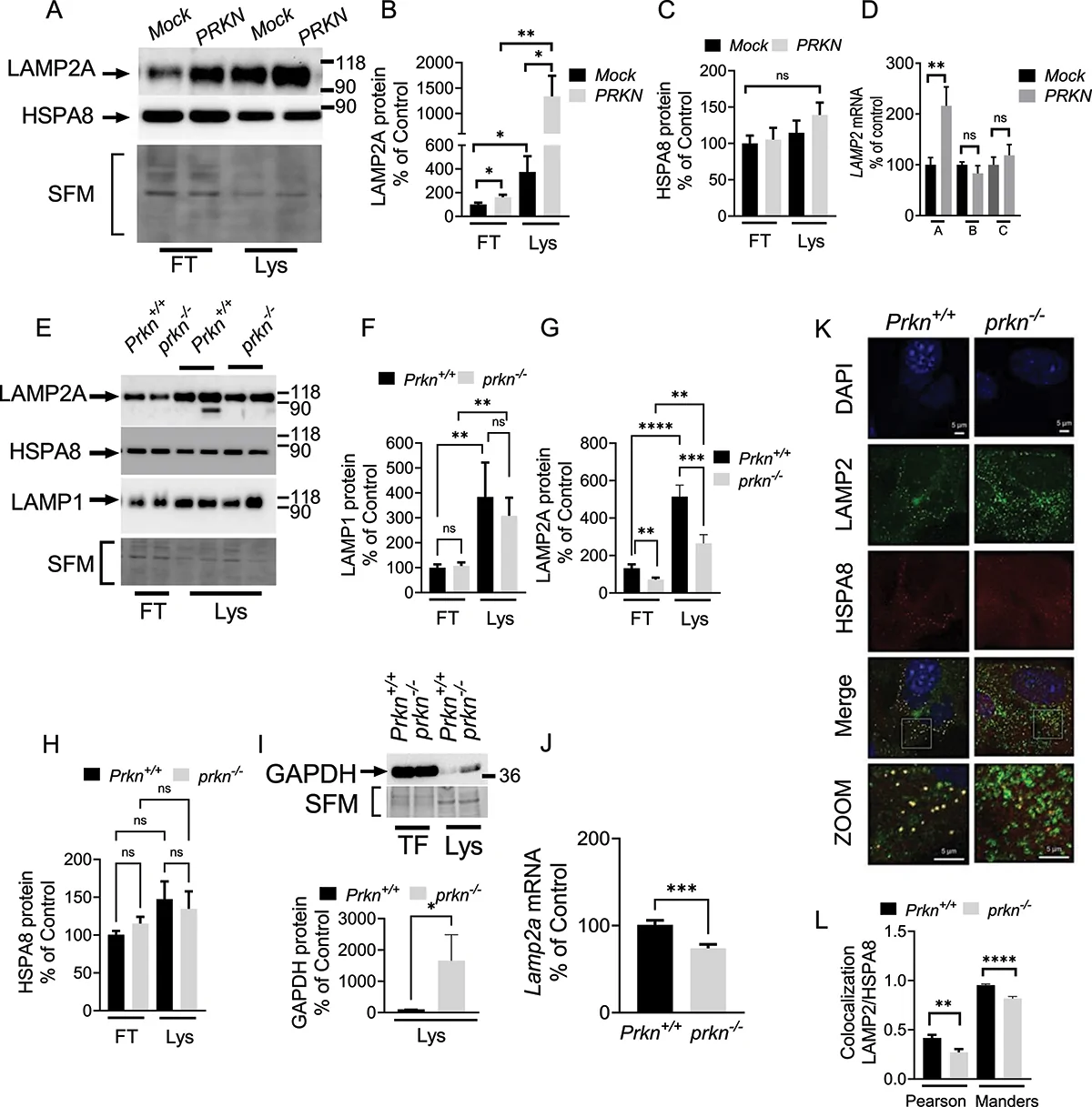
PRKN controls lysosomal LAMP2A expression *and CMA activity*. total fraction (FT) and lysosomal fractions (Lys) prepared from *Mock* and *PRKN*-transfected SH-SY5Y cells (A-D) or *Prkn*^+/+^ and *prkn^−/−^* fibroblasts (E-I) were assessed for LAMP2A (A, B, E and G), LAMP1 (E and F), HSPA8 (A, C, E and H) and GAPDH (I) protein expressions and for human *LAMP2A, LAMP2B, LAMP2C* (D) and mouse *Lamp2a* mRNA (J) levels. Proteins loading were assessed either by stain free membranes (SFM). Values are expressed as percent of FT in *Mock* (B-D) or *Prkn*^*+/*+^ (F-J) controls (taken as 100) and are the means ± SEM. Histological analysis of *Prkn^+/+^* and *prkn^−/−^* slices immunostained with anti-LAMP2 (green) and anti-HSPA8 (red) antibodies (G) (see methods). Nuclei are stained with DAPI (blue), merged images of all tree channels are indicated and zoom correspond to the squared zones indicated in merge images. (L) quantification of colocalization were analyzed by Pearson’s and Manders correlation coefficients. Scale bars: 10 μm. Bars represent the means ± SEM. *p ≤ 0.05; **p ≤ 0.01; ***p ≤ 0.001; ns: not statistically significant. Full gels are provided in data S3.

GBA1 participates in CMA-mediated degradation of SNCA [21] and was shown to modulate CMA [11]. Thus, we examined the putative role of PRKN in the regulation of CMA. First, we have analyzed the influence of PRKN overexpression or depletion on two specific key CMA players, namely, HSPA8, a chaperone protein involved in the recognition of KERFQ bearing proteins in the cytosol, and LAMP2A, a lysosomal membrane protein which promotes the translocation of target proteins for degradation in lysosomal lumen [22]. Since CMA activation requires the translocation of HSPA8 to the lysosome rather than modulation of its protein expression [23], and according to the key role of this cell compartment for CMA process, we examined the expression of LAMP2A and HSPA8 in whole SH-SY5Y homogenate, (total fraction [FT]) and isolated lysosomal (Lys) fractions upon PRKN modulation. PRKN overexpression increased LAMP2A protein (Figure 4(A,B), *N* = 12, t-test, 4 independent experiments performed in triplicate) and mRNA levels (Figure 4(D), *N* = 9, t-test, 3 independent experiments performed in triplicate), and appeared biological inert on HSPA8 expression (Figure 4(A,C), *N* = 9, t-test, 3 independent experiments performed in triplicate). Of interest, the PRKN-associated gene modulation of *LAMP2A* appeared isoform-specific since *LAMP2B* and *LAMP2C* were not modulated by PRKN (Figure 4(D), *N =* 9, Mann-Whitney test, 3 independent experiments performed in triplicate). The opposite phenotype was observed when comparing *prkn*^*-/-*^ *and Prkn*^*+/+*^ *MEF* cells. Thus, a reduction of LAMP2A protein levels (Figure 4(E,G), *N* = 8, t-test, 4 independent experiments performed in duplicate) and mRNA (Figure 4(J), *N* = 15, t-test, 5 independent experiments performed in triplicate) without affecting LAMP1 (Figure 4(E,F), *N* = 8, 4 independent experiments performed in duplicate) and HSPA8 protein levels (Figure 4(E,H), *N* = 8, t-test, 4 independent experiments performed in duplicate) was observed. Interestingly, Pearson and Mander’s correlation analysis indicated lower colocalization of HSPA8 and LAMP2 depicting a reduced number of CMA active lysosomes (Figure 4(K,L), *N* = 9, t-test, 3 independent experiments performed in triplicate) in *prkn*^*-/-*^ MEFs. Of interest, the highly increased lysosomal expression of the CMA substrate GAPDH (Figure 4(I), *N* = 5, Mann-Whitney test, 2 independent experiments performed in duplicate and triplicate, respectively), upon *prkn* gene knockout strengthens the conclusion that CMA was dependent/activated by endogenous PRKN. This conclusion also stands in SH-SY5Y cells. Thus, *PRKN* reduction decreased LAMP2A protein (Figure S4A and B, *N* = 12, Mann-Whitney test, 4 independent experiments performed in triplicate) and mRNA expressions (Figure S4C, *N* = 9, Mann-Whitney test, 3 independent experiments performed in triplicate) without affecting HSPA8 protein expression (Figure S4A and B, *N* = 12, Mann-Whitney test, 4 independent experiments performed in triplicate). Corroborating data obtained in MEFs, *PRKN* knockdown in SH-SY5Y cells also led to a reduction on LAMP2A and HSPA8 colocalization *in situ (*Figure S4D). This colocalization was illustrated by both Pearson and Mander’s coefficient (Figure S4E, *N* = 5 Mann-Whitney test, 2 independent experiments performed in duplicate and triplicate, respectively). Thus, this strengthens the conclusion that PRKN indeed activates CMA in human neuronal-like cells.

### Endogenous PRKN controls SNCA, GBA1 and CMA in the mouse brain

**Figure 5.**
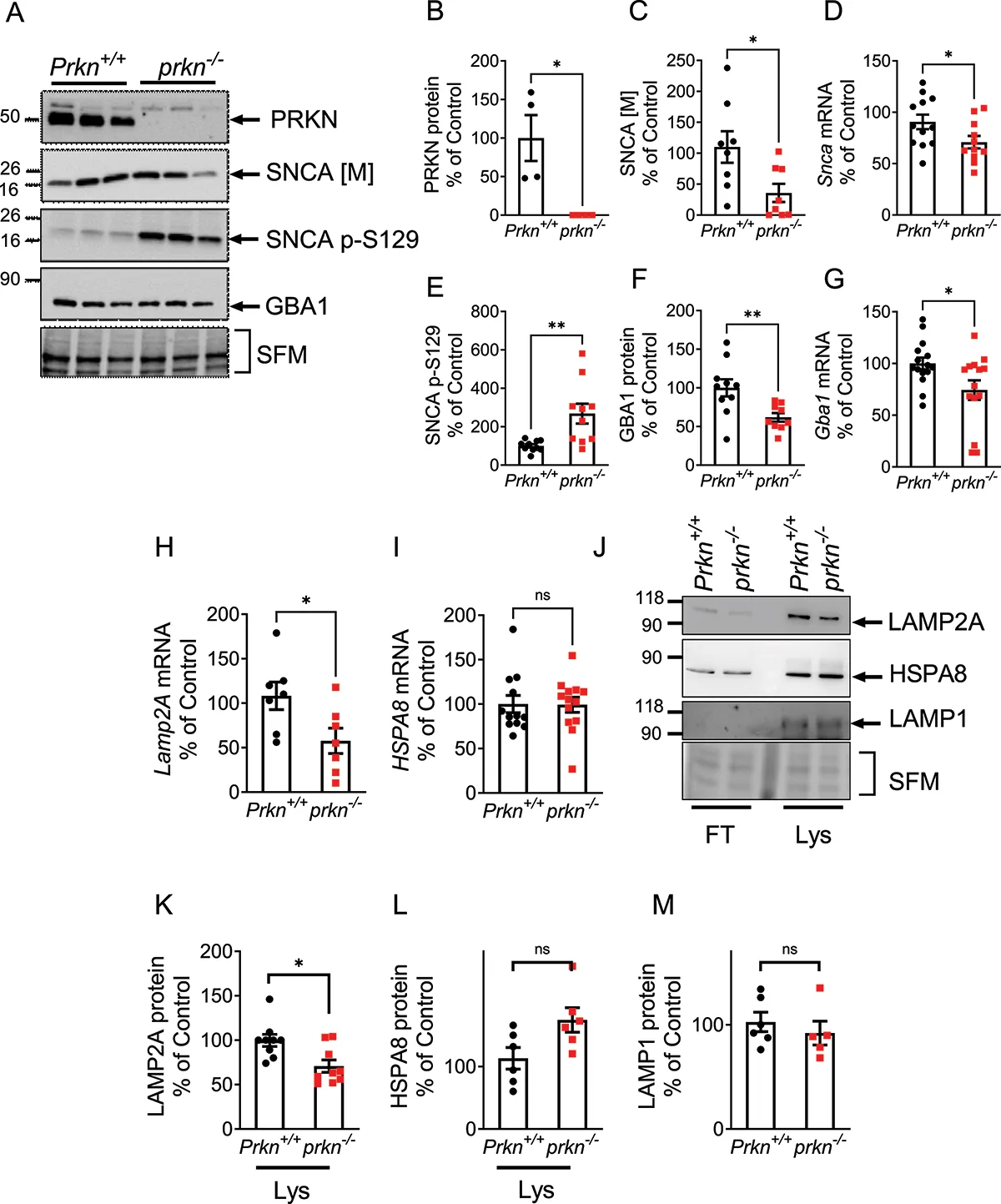
Proteins and mRNA expressions in the brain of *prkn^−/−^* mice. Nine-month-old *Prkn*^+/+^ and p*rkn^−/−^* whole brain mice samples were assessed for PRKN (A and B), SNCA [M] (A and C), SNCA p-S129 (A and E), GBA1 (A and F) protein expressions and *Snca* (D), *Gba1* (G), *Lamp2a* (H) and *Hspa8* (I) mRNA expressions. Protein loading was assessed either by stain free membranes (SFM). Bars are expressed as percent of control *Prkn^+/+^* mice (taken as 100) and are the means ± SEM. *p ≤ 0.05; **p ≤ 0.01; ***p < 0.001; ns: not statistically significant. (J-M) total (FT) and lysosomal (Lys) fractions were prepared from 9-month-old mice striatum and analysed for LAMP2A (J and K), HSPA8 (J and L) and LAMP1 (J and M) protein expressions. Bars are expressed as percent of control *Prkn^+/+^* mice (taken as 100) and are the means ± SEM. Full gels are provided in data S4.

Next, we explored the ability of endogenous PRKN to control SNCA [M] and SNCA p-S129 forms in the mouse brain. At nine months of age, brains from *prkn*^*-/-*^ knockout gene mice (see PRKN expression in Figure 5(A,B)) displayed decreased levels of SNCA monomeric protein (Figure 5(A,C), *N =* 8, t-test, 4 independent experiments performed in duplicate) and *Snca* mRNA (Figure 5(D), *N =* 12, t-test, 4 independent experiments performed in triplicate) expression while the levels of SNCA p-S129 were strongly increased (Figure 5(A,E), *N =*10, t-test, 5 independent experiments performed in duplicate). In agreement with our previous data and conclusions, *Prkn* gene deletion in mice also led to the reduction of GBA1 protein (Figure 5(A,F), *N =* 9, t-test, 3 independent experiments performed in triplicate) as well as *Gba1* (Figure 5(G), *N =* 15, t-test, 5 independent experiments performed in triplicate) and *Lamp2a* (Figure 5(H), *N =* 7, Mann-Whitney test, 2 independent experiments performed in duplicate and triplicate) mRNA levels while *Hspa8* (Figure 5(I), *N =* 9, Mann-Whitney test, 3 independent experiments performed in triplicate) mRNA expression remained unaffected. Of utmost importance, the analysis of LAMP2A, HSPA8 and LAMP1 in lysosomes prepared from *prkn*^*-/-*^ mice brain fully corroborated our conclusions, i.e a lowering of LAMP2A (Figure 5(J,K), *N* = 9, Mann-Whitney test, 3 independent experiments performed in triplicate) but not LAMP1 (Figure 5(J,M), *N* = 9, Mann-Whitney test, 3 independent experiments performed in triplicate) and HSPA8 (Figure 5(J,L), *N* = 9, Mann-Whitney test, 3 independent experiments performed in triplicate) expressions in absence of endogenous PRKN. Overall, these data support the fact that, *in vivo*, in a physiologically integrated context, the depletion of endogenous *Prkn* gene could well contribute to the lowering of SNCA monomeric and accumulation of neurotoxic SNCA p-S129 via the down-regulation of *GBA1* transcription and CMA-related SNCA degradation.

### PRKN controls paraquat-induced SNCA, GBA1 and LAMP2A alterations in a PD-like model

**Figure 6.**
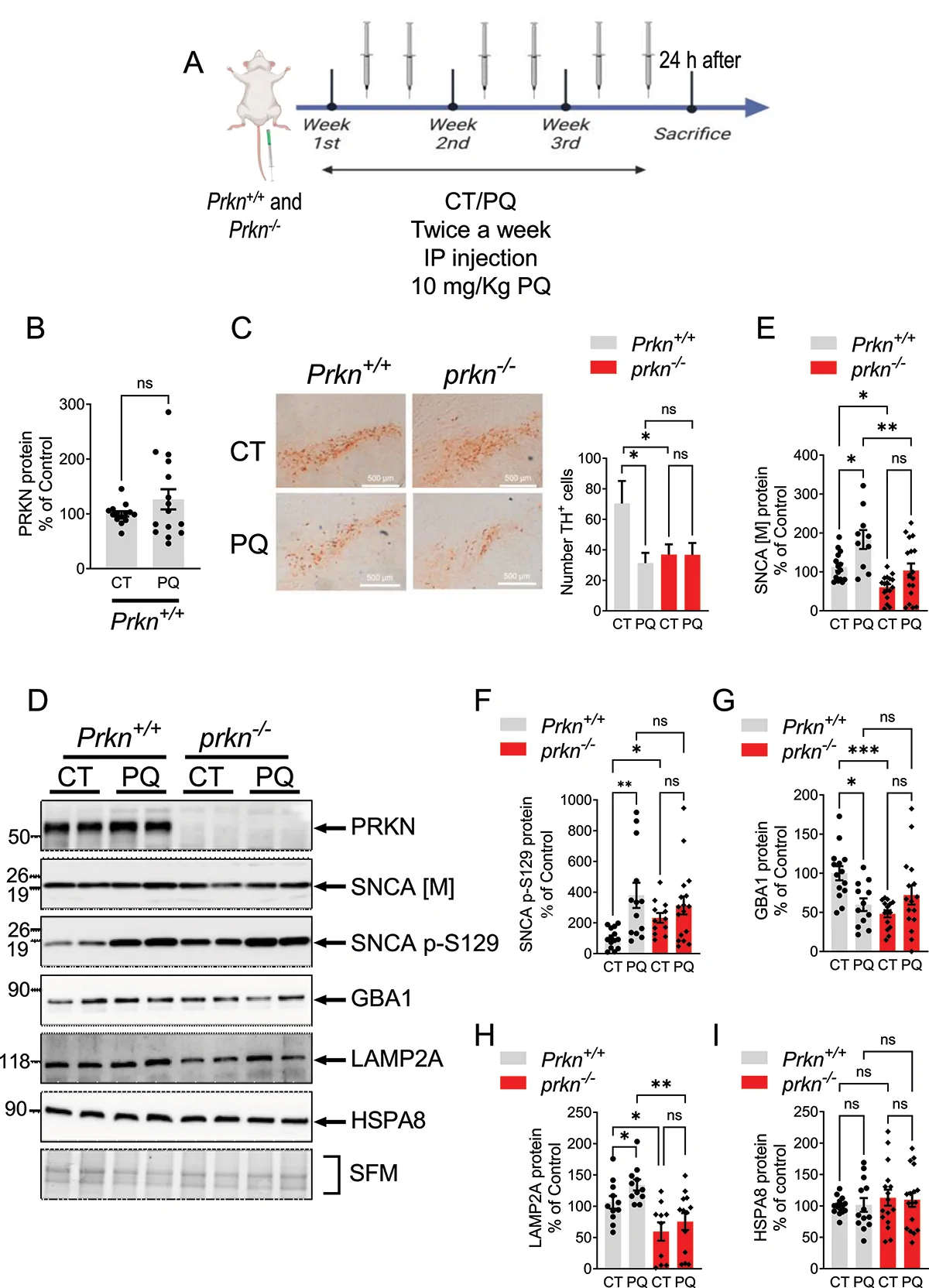
Paraquat-induced modulation of *Snca* and CMA in *prkn^−/−^* mice. (A) Schematic representation of paraquat (PQ) administration procedure in nine-month-old *Prkn^+/+^* and *prkn^−/−^* mice. Histological analysis (C) and quantification (D) of PQ-induced reduction of the number of tyrosine hydroxylase)-positive (TH^+^) cells in the striatum of the indicated mice. Scale bars: 500 μm. Bars correspond to the number of TH^+^-positive cells and are the means of 15 slices. Expressions of PRKN (B and D), SNCA [M] (D and E), SNCA p-S129 (D and F), GBA1 (D and G), LAMP2A (D and H) and HSPA8 (D and I) in the striatum of control (CT) and PQ-treated *Prkn^+/+^* and *prkn^−/−^* mice. Bars are expressed as percent of control (*Prkn*^+/+^, CT) (taken as 100) and are the means ± SEM. *p ≤ 0.05; **p ≤ 0.01; ***p < 0.001; ****p ≤ 0.0001; ns: not statistically significant. Stain free membranes (sfm) is provided to illustrate protein load. Full gels are provided in data S5.

We examined whether the above-delineated cascade was altered in a pharmacologically-induced PD condition. Paraquat (PQ) is an herbicide that has been associated to an increased risk of developing sporadic PD. Of note, this compound frequently used to mimic the disease in mice [24], was reported to modulate cellular PRKN localization, thereby impairing its transcriptional function [25]. Thus, we took advantage of this PQ-induced sporadic PD model to evaluate its effect on SNCA, GBA1 and LAMP2A in wild-type (*Prkn*^*+/+*^) and knockout (*prkn*^*-/-*^) transgenic mice. Figure 6 shows that PQ administration twice a week for three weeks (see experimental schedule in Figure 6(A)) did not affect PRKN protein expression (Figure 6(B), *N* = 15, t-test) but lowered *in situ* TH (tyrosine hydroxylase) expression and number of TH^+^ cells (Figure 6(C), *N =* 12, one-way ANOVA, Sidak’s multiple comparison test) in *Prkn*^*+/+*^ mice, thus confirming the PQ-induced loss of dopaminergic cells in our experimental procedure. PQ-treated *Prkn*^*+/+*^ mice display increased SNCA monomeric (Figure 6(D,E), *N =* 10–15, one-way ANOVA, Sidak’s multiple comparison test) and SNCA p-S129 (Figure 6(D,F), *N =* 10–15, one-way ANOVA, Sidak’s multiple comparison test) levels and decreased levels of GBA1 (Figure 6(D,G), *N =* 10–15, one-way ANOVA, Sidak’s multiple comparison test). In these wild-type mice, PQ induced a significant CMA activation, illustrated by enhanced LAMP2A expression (Figure 6(D,H), *N =* 10–15, one-way ANOVA, Sidak’s multiple comparison test) while HSPA8 expression was not significantly impacted (Figure 6(D,I), *N =* 10–15, one-way ANOVA, Sidak’s multiple comparison test). Of utmost importance, PQ-associated phenotypic alterations of the above-described proteins were all wiped out by the depletion of endogenous PRKN protein (Figure 6(D–I), compare CT and PQ red bars). This set of data suggests that PQ acted upstream to PRKN, likely impairing the control of its direct (SNCA and GBA1) and indirect (LAMP2A) transcriptional targets in mice.

### Analysis of PRKN, SNCA, GBA1 and CMA markers in the brains of human sporadic PD-affected brains and fibroblasts of PRKN mutation carriers

**Figure 7.**
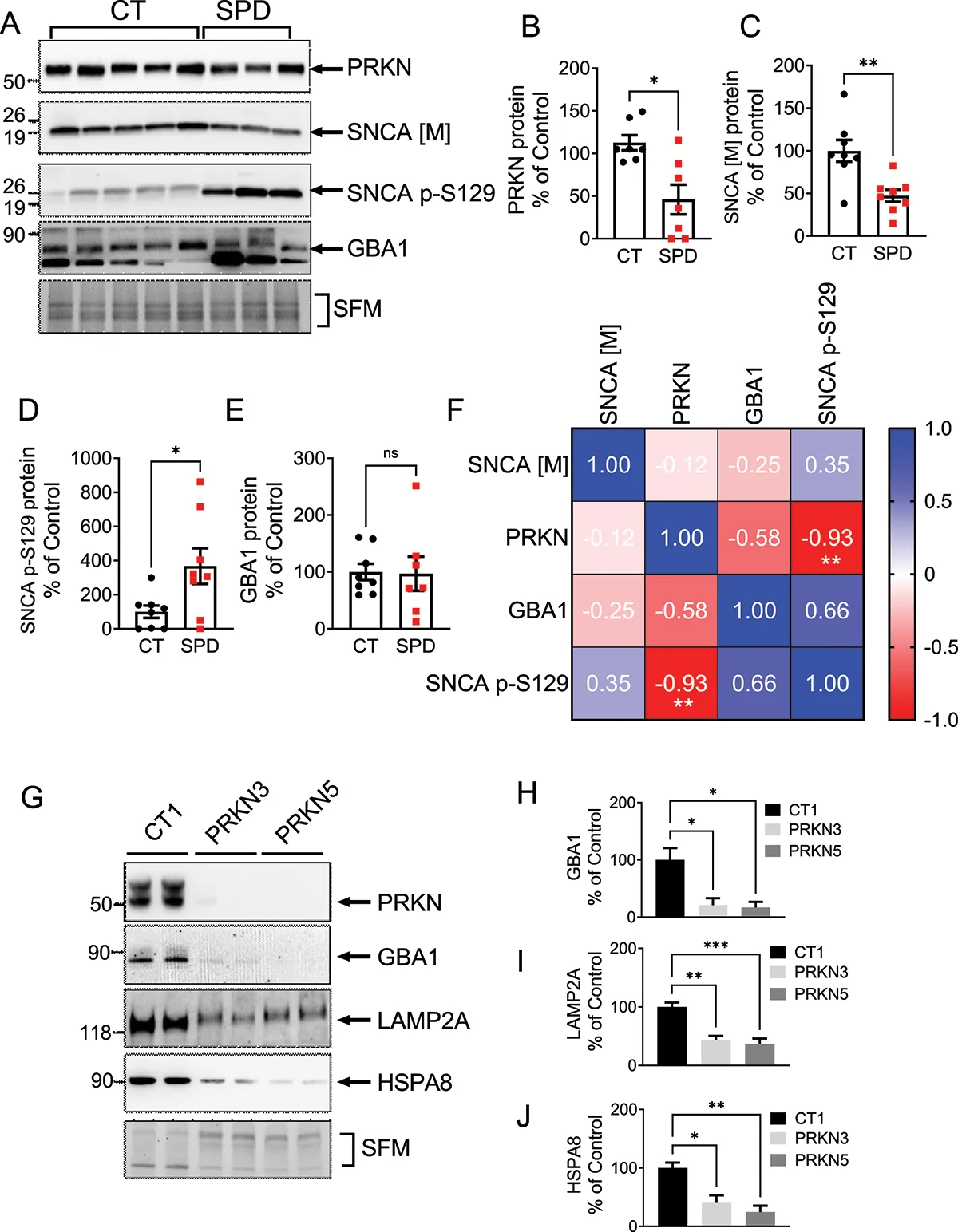
Analysis of PRKN-GBA1-SNCA cascade in the brain of sporadic PD patients and in human fibroblasts harboring familial *PRKN* mutations. *substantia nigra* of control (CT) and sporadic Parkinson disease (SPD) cases were analyzed for PRKN (A and B), SNCA [M] (A and C), SNCA p-S129 (A and D) and GBA1 (A and E) proteins expressions as described in methods. Pearson correlation analyses between PRKN (A and B), SNCA [M] (A and C), SNCA p-S129 (A and D) and GBA1 (A and E) protein expressions in sporadic pd brain samples (SPD) are shown in F. Fibroblasts of control and familial Parkinson disease (FPD)-linked to *PRKN* mutations (*PRKN3* and *PRKN5*) were analyzed for PRKN (G), GBA1 (G and H), LAMP2A (G and I) and HSPA8 (G and J). Bars are expressed as percent of control brains/fibroblasts (taken as 100) and are the means ± SEM. *p ≤ 0.05; **p ≤ 0.01; ***p < 0.001 and ns: non-significant. sfm: stain free membrane to illustrate protein load. Full gels are provided in data S6.

We evaluated the levels of PRKN, SNCA and GBA1 in the brains from patients with sporadic Parkinson disease (SPD). We observed a significant reduction of PRKN (Figure 7(A,B), *N =* 7, Mann-Whitney test) and SNCA [M] (Figure 7(A,C), *N =* 8, Mann-Whitney test) expressions, while SNCA p-S129 levels were increased (Figure 7(A,D), *N =* 8, t-test). However, one should note that the protein levels of GBA1 were not significantly regulated in our pathologic brain samples (Figure 7(A,E), *N=*8, Mann-Whitney test). Interestingly the examination of a Pearson correlation analysis between PRKN, SNCA [M], SNCA p-S129 and GBA1 protein levels in sporadic PD (SPD) brain samples indicated a strong and significative (Figure 7(F), Pearson r −0.93, *p* < 0.01) negative correlation between PRKN and pathogenic SNCA p-S129 protein levels. This set of data (Figure 7(A–F)) indicated that in SPD, a reduction of PRKN was indeed accompanied by alterations in SNCA [M] and SNCA p-S129 reminiscent of those observed in dopaminergic cells and experimental PD mouse models.

Finally, we compared the levels of SNCA, GBA1, LAMP2A and HSC70 in human fibroblasts from control individuals or individuals bearing *PRKN* mutations (*PRKN3* = [{872-?} {1083+?}del]; [125 G > C] and *PRKN5* = [δ {535-?}_{618+?}];[1321T > C]). SNCA [M] was not detectable in human fibroblasts (not shown) as described in [26], but interestingly, we observed a significant reduction of GBA1 (Figure 7(G,H), *N =* 5–8, Kruskal-Wallis test followed by Dunn’s multiple comparison test, 4 independent experiments performed in duplicate), LAMP2A (Figure 7(G,I), *N =* 8, Kruskal-Wallis test followed by Dunn’s multiple comparison test, 4 independent experiments performed in duplicate) and HSPA8 (Figure 7(G,J), *N =* 8, Kruskal-Wallis test followed by Dunn’s multiple comparison test, 4 independent experiments performed in duplicate) protein levels in both *PRKN* mutant carriers fibroblasts. This set of data suggests that the same PRKN-linked pathological cascade leading to a reduction of GBA1 activity and, probably as a corollary, pathogenic SNCA accumulation as well as CMA could occur in both sporadic and familial forms of PD and that PRKN could be seen as a key factor at the center of gravity of this human pathology.

## Discussion

PD is a multifactorial neurodegenerative disease that leads to a progressive loss of dopaminergic neurons of the *substantia nigra*. It has been widely established that the main histopathological hallmark of PD [27,28] consists in Lewy bodies intracellular inclusions filled of insoluble SNCA fibrils. SNCA is a key gene product which, when mutated, is associated with both familial and sporadic cases of PD [4,29].

The physical status of SNCA conditions its function and pathological spreading. Thus, monomeric SNCA is protective via the lowering of the pro-apoptotic tumor suppressor p53 [9] and this function is abolished by both PD-linked mutations or by toxins triggering PD-like pathology [7,9]. Of note, these conditions all trigger SNCA aggregation. Interestingly, it was also reported that in PD-like conditions, the aggregation of SNCA is linked to its oxidation and to the impairment of proteasomal activity [30]. Thus, the homeostasis of cellular SNCA is controlled by its proteolytic fate, which likely conditions its concentration-dependent propensity to aggregate and to spread.

Along with this view, SNCA protein levels, notably its phosphorylated aggregated toxic forms, are increased in PD-affected brains [31]. However, previous data indicated that *SNCA* mRNA levels appeared reduced in these brains [12–14]. Thus, to address this paradoxical dichotomy between SNCA protein and mRNA levels regulation, it is first important to study the mechanisms governing the transcriptional regulation *SNCA* and second, to delineate the mechanisms by which SNCA homeostasis is controlled in cells as well as in *in vivo* models of PD.

Eight lines of independent evidence demonstrate that PRKN is a direct regulator of *SNCA* and *GBA1* transcriptions and controls the CMA in cells, *ex vivo* in human fibroblasts, *in vivo in mice* as well as in sporadic PD-affected human brains. First, overexpressed PRKN transcriptionally increased directly SNCA neuroprotective monomers, reduced phosphorylated/aggregation prone toxic SNCA and physically interacted with murine and human *SNCA/Snca* promoters. Second, overexpressed PRKN transcriptionally increased directly *GBA1* transcription. Third, PRKN-mediated control of *Snca* promoters was abolished by deletion of identified *PRKN*-responsive elements. Fourth, PRKN modulated CMA activity notably via the modulation of LAMP2A levels but not HSPA8, *in vitro*, *ex-vivo* (mouse and human fibroblasts) and *in vivo*. This appeared isoform specific since *LAMP2B* and *LAMP2C* mRNA levels were not affected. Fifth, PRKN-mediated control of SNCA was abolished by *GBA1* depletion in HAP1 cells. Sixth, the effect of the PD-inducing toxin paraquat on SNCA, GBA1 and LAMP2A was fully prevented by *Prkn* gene depletion in mice. Seventh, wild-type PRKN influenced monomeric and phosphorylated SNCA in PD-affected human brain samples. Eighth, human fibroblasts harboring a PRKN mutation displayed drastically lower CMA response and GBA1 expression. This consistent network of evidence undoubtedly identifies PRKN as a key regulator of GBA1- and CMA-controlled SNCA homeostasis.

Several works have suggested an interplay between PRKN and SNCA in PD [32–37] but none have addressed the possibility of a direct transcriptional control of *SNCA* by PRKN. Our work supports the assumption that the PRKN protein reduction observed by us (Figure 7(A,B)) and others [25] in human sporadic PD brain samples may account for the decrease of *SNCA* mRNA [12–14]. As a corollary, this set of data strongly suggests that SNCA degradation could indeed be controlled by PRKN.

Mutated *GBA1* is considered as a main genetic risk factor for developing PD [38,39] and is involved in CMA-mediated degradation of SNCA [1]. Thus, we postulated that PRKN-mediated control of SNCA expression/degradation could involve, directly or indirectly, its GBA-linked CMA-associated degradation. Interestingly, supporting our hypothesis, we identified PRKN-responsive elements on the two promoters (*P1* and *P2*) of the gene [40,41]. We showed for the first time, by multiple approaches including ChIP assay analysis (see Figure 3), that, as is the case for *SNCA*, *GBA1* is a direct transcriptional target of endogenous PRKN that interacts physically with its *P2* promoter. Of importance, we establish that *GBA1* invalidation triggers an accumulation of SNCA monomers and that PRKN-mediated modulation of SNCA expression was fully GBA-dependent in HAP1 cells (Figure 3(O,Q)).

Since GBA1 and CMA function are intimately linked [11] and according to the CMA-linked contribution to SNCA clearance [1], one would expect PRKN to regulate CMA. We showed that it is indeed the case. PRKN controlled CMA response *in vitro, ex vivo* and *in vivo* via the modulation of LAMP2A expression. Although the role of PRKN in the control of mitophagy had been largely documented [42], its role in lysosomal function is poorly known. However, PRKN has been associated to lysosomes biogenesis by controlling the nuclear translocation of TFEB (transcription factor EB), which acts as master lysosomal gene regulator [43]. Our study is the first demonstration of the implication of PRKN in the control of CMA and delineates a molecular cascade linking PRKN to CMA-mediated GBA1-associated control of SNCA expression. This PRKN-mediated phenotype explains the apparent paradoxical observation of decreased mRNA and increased SNCA protein expressions linked to decreased CMA-mediated SNCA degradation and protease-resistant form of phosphorylated/aggregated SNCA.

Several lines of evidences suggest that the above-described functional interplay between PRKN and SNCA could be altered in PD condition. Thus, we have investigated the impact of paraquat, an herbicide used to mimic sporadic Parkinson disease in mice. Importantly, it was shown that paraquat displays the ability to trigger PRKN nitrosylation, and abolish its transcriptional activity via its sequestration in the cytosol [25]. We unraveled a paraquat-induced reduction of GBA1 accompanied by an increase of phosphorylated forms of SNCA mimicking the data obtained in *Prkn* gene depleted mice. Further, we showed that PQ-associated modulation of phosphorylated SNCA, GBA1 and LAMP2A protein expressions were all abolished by *PRKN* depletion, indicating that PQ deleterious effect in mice could be mostly accounted for by the molecular cascade delineated in our study (summarized in Figure S5). However, it should also be noted that paraquat treatment still triggered a faint but statistically significant increase of SNCA monomers expression in *PRKN* invalidated mice. This indicates that paraquat likely controls SNCA expression mostly by a PRKN-dependent mechanism. In this context, it has been shown that paraquat induces a blockade of HMGB1 (high mobility group box 1)-mediated SNCA autophagic degradation by interfering with the interaction between HMGB1 and BECN1 (beclin 1) in SH-SY5Y cells [44]. This could account for the minor PRKN-independent PQ-induced modulation of SNCA expression.

Finally, we have analyzed the regulation of SNCA by PRKN in brain necropsies from control individuals and patients with sporadic PD. We observed a significant reduction of PRKN and SNCA monomers accompanied by an increase of phosphorylated SNCA. Of note, we observed a negative correlation between PRKN and phosphorylated SNCA levels, supporting the functional relationship between loss of endogenous PRKN function occurring in PD and accumulation of aggregated SNCA. Importantly, corroborating a role of PRKN in CMA in control conditions and familial PD, we have shown that PRKN mutations drastically reduced LAMP2A and HSPA8 protein levels in human fibroblasts, thus mimicking the data obtained in *prkn* gene KO mice.

Nitrosylation process is increased in neurodegenerative diseases [45], and has been particularly well documented in PD where inducible nitric oxide synthase protein expression and activity as well as mRNA levels are drastically enhanced [46]. Further, PRKN is S-nitrosylated in oxidative stress conditions and levels of S-nitrosylated PRKN are drastically enhanced in PD [47]. As stated above, PQ can trigger PRKN nitrosylation. Moreover, PQ-induced nitrosylation of PRKN impairs its transcriptional function by sequestrating it in the cytosol and preventing its shuttling to the nucleus [25]. Overall, the post-translational modifications of PRKN occurring in PD conditions could trigger the loss of PRKN function at the origin of the decrease of both neuroprotective SNCA and its GBA1- and CMA-mediated degradation and thereby, contribute to the accumulation of aggregated SNCA in Lewy bodies inclusions in sporadic PD. Since extracellular SNCA oligomers were also shown to induce PRKN S-nitrosylation [48], this suggests the existence of a regulatory forward deleterious loop between PRKN nitrosylation and SNCA aggregation in PD.

Taken together our data identifies *SNCA* and *GBA1* as PRKN transcriptional targets and demonstrates the involvement of PRKN in CMA-driven GBA1-dependent degradation of SNCA. It unravels the PRKN transcriptional function as a key target for novel therapeutic strategies aimed at blocking its inactivating nitrosylation process taking place in PD. Further, it identifies PRKN as a putative modulator of CMA, a key dysfunctional process that contributes to the exacerbation of aggregation process characterizing most of proteinopathies. This work highlights the importance of PRKN transcriptional factors function in PD, reinforces its role in autophagy control and opens new treatments perspective for PD and likely other proteinopathies based on the modulation of PRKN TF function.

## Materials and methods

### Plasmid constructions

The psbBiGP vectors empty or containing human wild-type *PRKN* cDNA used in this study to generate stable cell lines have been described [19]. The human *PRKN* shRNA and its scrambled sequence have been described [49].

The wild-type *Snca* mouse promoter cloning has been described [50]. This sequence contains one putative *PRKN*-RE binding site (5’-GCCGGAG-3’) in position −1081/-1075 upstream to the translation start codon. The deletion of this motif has been obtained by PCR using the forward primer 5”-CTGGGGATCTTAGCTAGGCAAAGAGCCGGGACGC-3‘ and the reverse primer 5’- GCGTCCCGGCTCTTTGCCTAGCTAAGATCCCCAG-3.‘ The human *SNCA* promoter has been cloned using a preparation of human genomic DNA obtained from SH-SY5Y cells (QIA amp DNA mini kit; Qiagen, 51,304). This genomic DNA was used as a template in a PCR reaction with the forward primer: 5’-CGACGCGTGGAAATCCTGGAGAACGCCG-3‘ and reverse primer 5’-GAAGATCTGGCTAATGAATTCCTTTACACCACACTGG-3” containing MluI and BglII restriction enzyme sites, respectively. After purification, the *SNCA* promoter fragment (1043 bp) was digested by MluI and BglII enzyme and subcloned in the pGL3 basic vector (Promega, E175).

The wild-type *GBA1* human promoter 1 (- 2128 to − 1207 from ATG) has been amplified by PCR with human genomic DNA as template by using the forward primer 5’-CTAGCTAGCGTGTGCCACTCCCGCTAAATC-3’containing a NheI site and the reverse primer 5’-CCCAAGCTTAGACCACAGGGGTTCCAGAGT-3' containing a HindIII site. After amplification, the purified and digested fragment have been subcloned between the NheI and Hind III site of the pGL3 basic plasmid. Similarly, to human *GBA1* promoter *1*, wild-type *GBA1* promoter 2 (- 5875 to − 4720 upstream the ATG) was amplified by PCR with the forward 5‘-CTAGCTAGCTTTCAGTGAGCACCCAATCCC-3’and the reverse 5’-CCCAAGCTTGAGAAAAGCAGCCCTGGGGAG-3‘ primers containing respectively a NheI and HindIII sites and subcloned in the pGL3 basic vector. The human *GBA* promoter *2* deleted of the putative PRKN-RE (5’-CTCCGGC-3”) in position − 4857 to − 4851 from ATG was obtained by PCR using the forward primer 5”- CTCGCTCTCGCTCTCTCTCTCTCGCCAGCGACACTT GTTCGTTC-3‘ and the reverse primer 5’-GAACGAACAAGTGTCGCTGGCGAGAGA GAGAGAGCGAGAGCGAG-3.” All the constructs appearing in Table 1 have been validated by full sequencing.

**Table 1.** List of human and mouse primers used in real-time quantitative PCR.

Name	Forward sequence primer	Reverse sequence primer
Human *PRKN*	5’-aaaaccaccaagccctgtc-3’	5’-tgcggacacttcatgtgc-3’
Human *SNCA*	5’-catggatgtattcatgaaaggact-3’	5’-tccttggttttggagcctac-3’
Human *GBA1*	5’-tcctaccctcgccaacagta-3’	5’-gctgcttctgggtctgtca-3’
Human *HSPA8*	5’-tttttgtggcttccttcgtt-3’	5’-tcccttggacatggttgc-3’
Human *LAMP2A*	5’-caacaaagagcagactgtttcag-3’	5’-gcactgcagtcttgagctgt-3’
Human *GAPDH*	5’-agccacatcgctcagacac-3’	5’- gcccaatacgaccaaatcc-3’
Human *TBP*	5’-attggtgttctgaataggctgtg-3’	5’-gaacatcatggatcagaacaacag-3’
Mouse *Prkn*	5’-gcatcccttgcatagcgtg-3’	5’-ggaagaccaggacagggctc-3’
Mouse *Snca*	5’-tggcagtgaggcttatgaaa-3’	5’-gcttcaggctcatagtcttgg-3’
Mouse *Gba1*	5’-gaccaacgcttgctgctac-3’	5’-acagcaatgccatgaacgta-3’
Mouse *Hspa8*	5’-ggaatacaaaggggagacaaaa-3’	5’-acagcgttggtaacggtct-3’
Mouse *Lamp2a*	5’-gcccctctgggaagttcttatatgtgc-3’	5’-tgaggtcagagtcagcagcacattc-3’
Mouse *Gapdh*	5’-aagagggatgctgcccttac-3’	5’-ccattttgtctacgggacga-3’
Mouse *Tbp*	5’- ggggagctgtgatgtgaagt-3’	5’-ccaggaaataattctggctcat-3’

### Human brain samples

Human *substantia nigra* samples were obtained from brains collected in a Brain Donation Program of the Brain Bank “Neuro-CEB” run by a consortium of patients Associations: CSC (cerebellar ataxias), Foundation ARSEP (research on multiple sclerosis), France Parkinson and “Vaincre Alzheimer Foundation.” The consents were signed by the patients themselves or their next of kin in their name, in accordance with the French Bioethical Laws. The Brain Bank Neuro-CEB (BB-0033–00011) has been declared at the Ministry of Higher Education and Research and has received approval to distribute samples (agreement AC-2013–1887). A total of nine controls and seven sporadic Parkinson disease patients were examined. The group of control samples is composed of five patients who have Alzheimer disease-like lesions (males nº 8730, 82 years-old and nº 7197, 85 years old and females nº 6283, 83 years-old, nº 6658, 93 years-old and nº 7024, 76 years-old), three amyotrophic lateral sclerosis not associated with Parkinson disease pathology patients (males nº1811, 55 years-old and nº 1822, 64 years-old; nº1821, female, 68 years-old) and one age-matched healthy patient (male nº1811, 61 years-old). The group of sporadic Parkinson disease patients is composed of three patients with AD-like lesions (nº4291, female, 72 years-old; two males nº8418, 82 years-old and nº 7743, 72 years-old) and four without Alzheimer disease lesions (males nº 4489, 75 years-old, nº5193, 75 years-old and nº 8460, 66 years-old; female nº 4513, 77 years-old). The mean *post-mortem* delay was 28.7 ± 15.4 h. The samples were homogenized and lysed as described below for mice brain samples.

### In vitro cellular models and transfections

SH-SY5Y human neuroblastoma cells were purchased from ATCC (CRL-2266^™^) and cultured at 37°C in 5% (vol:vol) CO_2_ in Dulbecco’s modified Eagle’s medium (DMEM; [Sigma-Aldrich, D6546]) supplemented with 10% of heat-inactivated fetal calf serum (FCS; [Dutcher, S1900-500]) containing 100 U/ml penicillin (Gibco, 15,140–122) and 550 µg/ml streptomycin (Life Technologies, 15,140,122). These cells were genetically manipulated to either stably overexpress or deplete PRKN by transposon and shRNA delivery approaches, respectively. To obtain SH-SY5Y cells depleted of PRKN (shRNA *PRKN*), cells were transfected with either scramble (*SC*) or shRNA *PRKN* vectors (sequences referenced in [49] and described in plasmids construction section) with Lipofectamine 2000 reagent (Invitrogen, 52,887) according to manufacturer instructions. Twenty-four h after transfection, cells were treated for one week for selection with 1 mg/ml geneticin (MP Biomedicals, 158,782) before being used for experiments. Polyclonal SH-SY5Y cells overexpressing low levels of PRKN were obtained by co-transfection of 10 ng of pSBbi bearing wild-type PRKN together with 5 ng of the pSB100X transposase (CMV(CAT)T7-SB100) provided by Dr. Z. Izsvak [51] (Addgene, 34,879) and 985 ng of pcDNA3 vector in 6-well plates using the Lipofectamine™ 2000 transfection reagent. Twenty-four h after transfection, cells were treated with 1 μg/ml puromycin (Sigma, P8833) and selection was carried out for one week. Cells were trypsinized and Mock and PRKN polyclonal cell populations were maintained with the corresponding selection antibiotic.

Human haploid (HAP1) cell lines invalidated by CRISPR-Cas9 approaches for the *SNCA* (Horizon, HZGHC003210c010) and *GBA1* (Horizon, HZGHC002786c001) genes were purchased from Horizon Discovery and cultivated at 37°C and 5% (vol:vol) CO_2_ in Iscove’s Modified Dulbecco’s Medium (IMDM; [Sigma-Aldrich, 12,440,053]) supplemented with 10% of heat-inactivated FCS (Dutscher, S1900-500) containing 100 U/ml penicillin (Gibco, 15,140–122) and 550 μg/ml streptomycin (Life Technologies, 15,140,122). In a subset of experiments, HAP1 cells were transiently transfected with 2 *μ*g of pSBbi PRKN vector in 6-well plate using Lipofectamine^TM^ 2000 reagent (Invitrogen, 52,887). After twenty-four h, cells were recovered for further experiments.

### Ex-vivo mouse and human cellular models

Primary cultured mouse embryonic fibroblasts (MEFs) were derived from mice kindly provided by Dr. Corti (ICM, Paris, France) [52], were prepared from *Prkn*^+/+^ and *prkn*^−/−^ C57BL/6 transgenic mice, as extensively described in [53]. Briefly, MEFs were obtained from mouse embryos at day 16 of gestation. The female mouse was euthanized by cervical dislocation and the embryos were recovered and dissected by removing heart, liver, head and all bloody tissues. The remaining parts of the fetuses were sliced in thin pieces, dissociated by ice-cold trypsin-EDTA (0.25%, [Sigma-Aldrich, 25,200,056]) and incubated overnight at 4°C. One day after, the tissue pieces were incubated for 30 min at 37°C in a water bath. Cell suspensions were obtained by multiple vigorous pipetting with medium (DMEM [Gibco, 41,965–039], 10% heat-inactivated FCS [Dutscher, 500105M1M], 100 U/ml penicillin [Gibco, 15,140–122] and 550 ug/ml streptomycin [Gibco, 15,140–122])., then plated in 10 cm tissue culture dishes in a proportion of one embryo/five dishes and cultured at 5% of CO_2_ and 37°C.

Human fibroblasts carrying PRKN mutations (PRKNRN3 = [(872-?)_(1083+?) del];[125 G > C] and PRKNRN5 = [(535-?)_(618+?) del];[1321T > C]) were provided by Dr. Olga Corti [54]. They were obtained from skin biopsies from PD patients and age-matched healthy individuals following routine clinical procedures, with previous written informed consent and the approval of the local ethics committee (“Comité de Protection des Personnes, Ile de France,” CCPPRB Paris-Necker under n° 04–06-08 MS8). They were cultured at 5% of CO_2_ and 37°C in Ham’s F-10, 15% of heat-inactivated FCS, penicillin (100 U/ml) and streptomycin (550 ug/ml).

### Promoter activity analysis

SH-SY5Y overexpressing or depleted of PRKN and primary cultured *Prkn*^*+/+*^ and *prkn*^*-/-*^ MEF cells were seeded in six-well plates and co-transfected with 1.2 µg of different luciferase-coupled promoter constructions described above and 0.8 µg of β-galactosidase control vector (Promega, E1081) by means of Lipofectamine^TM^ 2000 (Invitrogen, 52,887). Twenty-four h later, cells were lysed in reporter lysis buffer (Promega kit, E1500,) then 20 µl of cells extracts were transferred to a 96-well plate and luminescence signal analyzed in a microplate reader (Varioskan, Thermo Fisher Scientific, Waltham, USA). β-galactosidase activity analysis was performed to normalize promoter transfection efficiency by means of E200 kit (Promega kit, E200) according to manufacturer conditions in Varioskan microplate reader. Luciferase signals were normalized by both β-galactosidase activity and Bradford (Bio-Rad, 5,000,006) protein concentration measurements.

### mRNA extraction and real-time quantitative PCR

SH-SY5Y and primary MEF cells were seeded in 6-well plates. Forty-eight h later, the RNAs were extracted using RNeasy Mini Kit (Qiagen, 74,004) according to manufacturer instructions. RNAlater (Qiagen, AM7024) tissue or powder aliquots from stabilized mice brain (one hemisphere, striatum) were homogenized using MagNA Lyser Green beads tubes (Roche, 03358941001) via MagNA Lyser instrument (Roche, 03358976001) for 45 s at 4487 g in RNA extraction lysis buffer, then RNA extraction was performed using RNeasy Plus Universal Mini kit (Qiagen, 73,404) manufacturer’s instructions. A total amount of 2 *μ*g of RNA was reverse-transcribed in cDNA using Oligo-dT primer (Promega, C11001) and reverse transcriptase (Promega, A5002) following manufacturer’s guidelines. Real-time PCR was performed via Rotor-Gene 6000 (Qiagen, 9,001,862) apparatus, using SYBR Green detection protocol (Roche, 04887352001). The mRNA expression levels of *PRKN (Prkn)*, *SNCA (Snca)*, *GBA1 (Gba1)*, *LAMP2A (Lamp2a)* and *HSPA8* (*Hspa8*) genes in human (mouse) samples were normalized by *TBP* (*Tbp*) and *GAPDH (Gapdh)*. mRNAs levels were analyzed via Rotor Gene 6000 software (www.corbettreresearch.com version 1.7 for windows, software, US). All primers were designed by primer 3 software (https://primer3.ut.ee/version 4.1 for windows, software, Estonia), validated at Primer-Blast (NCBI) and are described below in Table 1.

### Western blot analyses

SH-SY5Y overexpressing or depleted of PRKN and primary cultured *Prkn*^*+/+*^ and *prkn*^*-/-*^ MEF cells were seeded (500000 and 200,000 for SH-SY5Y and MEF cells, respectively) on 6-well plates. Forty-eight h after, the cells were homogenized in lysis buffer (Tris-HCl 10 mM [Euromedex, 26–128-3094-B], pH 7.5 containing 150 mM NaCl [Sigma, S3014], 0.5% Triton X-100 [TX; Sigma, T8787], 5 mM EDTA [ethylenediaminetetraacetic acid; Sigma, 1.37004.1000], 10% protease inhibitor [Sigma, P8340], 5 µM sodium fluoride [Sigma, S7920] and 1 mM sodium orthovanadate [Sigma, 13,721–39-6]). One fresh brain hemisphere was homogenized in lysis buffer described above using MagNA Lyser Green beads tubes (Roche, 03358941001) via MagNA Lyser instrument (Roche, 03358976001) for 45 s at 4487 g. Frozen brain hemisphere was mechanically triturated and homogenized in the previously described lysis buffer in a potter apparatus. The analysis of SNCA oligomers was performed according to the protocol of Kim et al. [55] with minor modifications. In brief, cells were cross-linked with disuccinimidyl suberate (DSS; [Thermo Fisher Scientific, A39267]) according to manufacturer’s protocol, homogenized in Triton X-100 (TX)-soluble buffer (50 mM Tris-HCl, pH 8, 150 mM NaCl [Sigma, S3014], 1% Triton X-100 [Sigma, T8787], 5 mM EDTA [Sigma, 1.37004.1000], 10% protease inhibitor [Sigma, P8340], 5 µM sodium fluoride [Sigma, S7920] and 1 mM sodium orthovanadate [Sigma, 13,721–39-6]) and centrifuged at 20,000 g, 4°C for 45 min. Cells and mice samples were sonicated (40–50 pulses for 16 seconds for cells and 2 × 16 seconds for mice samples) and protein levels dosed by Bradford assay (Bio-Rad, 5,000,006). Ten to fifty μg of proteins were separated by SDS-PAGE (140 V, 400 mA and 150 W) on 10–12% TGX Stain-free fast cast acrylamide gels (Bio-Rad, 1,610,183, 1,610,185). Then, gels were stain-free activated on ChemiDoc™ Imaging System (Bio-Rad, 12,003,154) and proteins transferred (10 min, 2.5 A and 25 V) to nitrocellulose membranes (Bio-Rad, 1,704,271) by means of a Trans-blot turbo semi-dry transfer system (Bio-Rad, 1,704,150). Membranes were blocked for one hour in 5% skimmed milk dissolved in phosphate-buffered saline (PBS; Euromedex, ET330-A), except for the analysis of SNCA [M], in which the membrane was blocked for 30 min in 0.4% PFA (EMS, 15,744) dissolved in 5% skimmed milk and then 30 additional min in skimmed milk. The analysis of SNCA p-S129 was performed exactly as described by Lazaro et al. [56].

Transferred proteins were immuno-blotted overnight with rabbit anti-monomeric SNCA (Cell Signaling Technology, 2642), rabbit anti-phopho S129 SNCA (Cell Signaling Technology, 23,706), mouse anti-oligomeric and monomeric SNCA (BD Biosciences, 610,787), rabbit anti-GBA1 (Sigma-Aldrich, G4717), rabbit anti-LAMP2A (Invitrogen Thermo Fisher Scientific, 512,200), rat anti-LAMP2 (Santa Cruz Biotechnology, sc-19991), mouse anti-HSPA8/Hsc70 (Santa Cruz Biotechnology, sc-7298), mouse anti-PRKN (EMD Merck Millipore, MAB5512) mouse anti-ACTB/β-actin (Sigma-Aldrich, A5136), human LAMP1 (Santa Cruz Biotechnolog, sc20011), mouse LAMP1 (BD Pharmingen, 553,792) and anti-GAPDH (Sigma-Aldrich, MAB374 clone 6C5). Anti-TUBB/tubulin (Sigma, T4026).

Immunological complexes were revealed with either anti-rabbit, anti-mouse and anti-rat IgG-coupled peroxidase antibodies (Jackson ImmunoResearch, AB_2337943, AB_2338518, AB_2338141) by the electrochemiluminescence bright quantum and sirius detection reagents (Advansta, K12042D20 and K12043D20). Chemiluminescence was recorded using a luminescence image analyzer ChemiDoc™ Imaging System (Bio-Rad, 12,003,154) and quantifications of non-saturated images were performed with the Image lab 6.0.1 software for Windows. The utilization of stain-free imaging (gel or membrane) allows normalization of the protein levels of interest protein according to the total protein load in each lane. This normalizing strategy is particularly useful when the analysis of classical protein load markers (TUBB/tubulin, ACTB/actin) have a molecular weight comparable to the one of the proteins of interest. Thus, all the blots provided were normalized by either stain free membranes (SFM; Bio-Rad, 12,003,154) or TUBB/tubulin or ACTB/actin levels when possible. Full gels of main and supplementary figures are provided in **DATA S1-S9**.

### Lysosome preparation

The isolation of lysosomes was performed by means of a Lysosome Isolation Kit (Invent Biotechnologies, LY-034) according to manufacturer conditions or by validated methods as briefly described thereafter. Thus, cells were harvested by centrifugation (1,000 g, 3 min, 4 °C), lysed, homogenized with a syringe (26 G needles; [Agani Terumo, AN *2613R1]) in 200 μl of 20 mM HEPES buffer, pH 7.4, (Sigma, H3375), 1.5 mM MgCl_2_ (Sigma, M8266) , 1 mM EDTA (Sigma, 1.37004.1000 number), 1 mM EGTA (ethylene glycol-bis(β-aminoethyl ether)-N,N,N‘,N‘-tetraacetic acid; [Sigma, 67–42-5]), 1 mM DTT (dithiothreitol; [Sigma, 3483–12-3]), protease inhibitors cocktail [Sigma, P8340]. Aliquots were made and correspond to total cell lysates. To obtain the membrane fraction the remaining lysates were incubated on ice for 15 min, centrifuged at 850 g for 5 min at 4°C. The supernatant was centrifuged again at 20,000 g for 1 h 30 min at 4°C the pellets obtained containing the membrane were resuspended in RIPA buffer (50 mM Tris, pH 7.4 [Euromedex, 26–128-3094-B], 150 mM NaCl, 1 mM EDTA [Sigma, S3014], 1% Triton X100 [Sigma, 1.37004.1000], 0.5% sodium deoxycholate [Sigma, D6750], 0.1% SDS from a 20% solution [MP, SDS20002], protease inhibitors [Sigma, P8340]) and both whole cell lysates and membrane fractions were submitted to western blot analysis.

### In vivo studies

*Prkn*^*+/+*^ and *prkn*^*-/-*^ C57BL/6 mouse [52] were housed with a 12-hours light/dark cycle and were given free access to food and water. Nine-month-old male mice were sacrificed by cervical dislocation and the brains were recovered and stabilized in RNAlater (Qiagen, AM7024) for 24 h before freezing at −80°C for subsequent protein and mRNA levels analyses.

In a subset of experiments, mice were previously submitted to a pharmacological treatment with paraquat according to a protocol validated by European Communities Council Directive (86/609/EEC) and local French regulation. Thus, 9-month-old male mice were injected intraperitoneally (i.p.) twice a week for 3 weeks with 10 mg/kg of paraquat (Sigma-Aldrich, 36,541) or vehicle (saline). Twenty-four h after the last injection, a subset of mice was sacrificed by cervical dislocation for biochemical analysis. In brief, mice brains were recovered and stabilized in RNAlater for 24 h and the striatum dissected and frozen at −80°C. Then, frozen striata were triturated into powder and aliquoted for further analysis by western blot. A second subset of mice (3 mice per experimental condition) was deeply anesthetized with pentobarbital and submitted to intracardiac perfusion of PBS and 4% paraformaldehyde (PFA). Brains were recovered and post-fixed in PFA for 24 h and then conserved in PBS at 4°C for immunohistochemical analysis.

### Enzymatic GBA1 assay

GBA1 activity was performed as described in (9) with minor modifications. In brief, SH-SY5Y overexpressing or depleted for PRKN (500 000 cells) and *Prkn*^*+/+*^ or *prkn*^*-/-*^ MEF (200 000 cells) cells were seeded on 6-well plates. Forty-eight h later, cells were recovered and homogenized in lysis buffer (50 mM citric acid, [Rectapur, 20,273.292], 176 mM K_2_HPO_4_ [Sigma, 7758–11-4], 10 mM sodium taurochalate [Sigma, 145–42-6], 0.01% Tween-20 [Sigma, P1379] at pH 5.9). Lysed cells (seeded in 96- well black plates [Greiner Bio-One, 6750–76]) were incubated for 30 min on ice and 5 μl of samples were assayed in 93 μl of activity assay buffer (32% lysis buffer, containing 2.5 mM GBA1 substrate 4-methylumbelliferyl β-D-glucopyranoside [Sigma-Aldrich, M3633] in the presence or in the absence of 2.5 mM of GBA1/β-glucosidase inhibitor, conduritol B epoxide (CBE; Sigma-Aldrich, HY-100944). The fluorescence (excitation of 355 nm and emission of 460 nm wavelengths) was recorded in a Varioskan microplate reader (Thermo Fisher Scientific, Waltham, USA). The GBA activity is considered as the CBE-sensitive specific activity.

### Colocalization studies

SH-SY5Ycells (100 000) overexpressing or depleted of PRKN and *Prkn*^*+/+*^ or *prkn*^*-/-*^ MEF were seeded on 12-well plate with 18-mm cover glass (Marienfeld, 0111580). Forty-eight h after, cells were fixed for 30 min in 4% PFA, (EMS, 15,744), subsequently alkynylated for 10 min in 50 mM NH_4_CL (Sigma, 12,125–02-9) then permeabilized for 10 min in 0.1% Triton X-100 (Sigma, T8787). Non-specific sites were blocked for 20 min with PBS 0.05% Tween-20 (Sigma, P1379) containing 5% BSA (Sigma, A9747). Cover slips were incubated for 2 h with a mix of primary antibodies (anti-HSPA8 and anti-LAMP2) at room temperature, rinsed and incubated for 1 h with a mix of secondary antibodies (donkey anti-mouse Alexa Fluor-647, for HSPA8 and goat anti-rat Alexa Fluor-594, for LAMP2A; Invitrogen, A31571 and A11007, respectively). Slices were counterstained with DAPI (Thermo Fisher Scientific, 62,248). Images were taken with a Laser Scanning Confocal Microscope (Carl Zeiss, LSM780, Oberkochen, Germany) using an HCX PL APO 63×/1.4–0.6 (Leica, 506,192, Wetzlar, Germany) oil immersion objective and managed with our OMERO-MICA database [57]. Colocalization analysis was performed with ImageJ/Fiji using Pearson correlation coefficient obtained with the JACoP plug-in [58].

### Mice immunohistochemical analysis of TH

After perfusion (see above), twelve mice brains were sliced in 40-*μ*m free-floating coronal sections using a microtome HM 650 V (Thermo Fisher Scientific, Waltham, USA), cryoprotected by cold anti-freeze solution (3% NaH_2_PO_4_ [PROLABO, 28 026292], 10% Na_2_HPO_4_, [Sigma, 71,500], 20% glycerol [VWR chemicals, 24,388.295], 30% ethylene glycol [Carlo Erba Reagents, 346,501]) and water then stored at 4°C until histological staining. From each mouse, 6 sections with intervals of 4 slices were selected starting from caudal to rostral position of *substantia nigra* to cover the entire region. Sections were washed in PBS, permeabilized in 0.5 TX and blocked in 3% H_2_0_2_ 30% (Sigma, 216,763) solution to prevent nonspecific binding. Sections were incubated overnight with anti-tyrosine hydroxylase (GENETEX, GTX113016), washed, incubated for 2 h at room temperature with HRP-conjugated anti-rabbit antibodies, (Jackson, AB_2337943) and incubated with 3,3’-diaminobenzidine tetrachloride (DAB; [Vector labs, SK-4105). Images acquisition were performed in a light microscope (DMD108, Leica Microsystems, Nanterre, France) through a N PLAN 10×/0.25 objective. The counting of tyrosine hydroxylase positive neurons (TH+ neurons) in the *substantia nigra par compacta* was performed with a ImageJ/Fiji macro-program [59]. After a segmentation using an Auto Local Threshold of the green component of the RGB image, the holes were filled and a binary opening was applied before object counting. This macro was applied to the images stored in the OMERO-MICA database using the Batch OMERO plugin [60]. The quantification of TH+ neurons counts were expressed in means ± SEM.

### Chromatin immunoprecipitation

The DNA-protein interactions were performed with CUT&RUN Kits (Cell Signaling Technology, 86,652) according to supplier instructions and as described in [19]. The interaction of endogenous and overexpressed PRKN with human promotor regions of *SNCA and GBA1* was examined in control or SH-SY5Y stably overexpressing wild-type PRKN. PRKN immunoprecipitation was performed with anti-PRKN antibody (Merck Millipore, MAB 5512). The primers targeting the human promoter regions of *GBA1* (forward: 5’-gctctctctcgctcgctctct-3’ and reward: 5’-aacgaacaagtgtcgctggcg-3’) and *SNCA* (Site 1: forward: 5'-ggaaatcctggagaacgccgga-3‘ and reward: 5’-cgggtcctcatggcagca-3‘; site 2: forward: 5’-ccttctccacaagggctgagag-3‘ and reward: 5’-ctccatggagcatcctcgcg-3‘ and site 3: forward: 5’-ctgtctgcccgctctcttgg-3’and reward: 5”-aacgaacaagtgtcgctggcg-3’) encompassing validated *PRKN*-RE were purchased from Eurogentec (Seraing, Belgium). The percent of input was calculated and used as reference as described previously [19].

### Statistical analysis

Statistical analysis was performed with GraphPad Prism software (www.graphpad.com version 9.00 for Windows, software, San Diego, California, USA). All groups of samples were submitted to normality tests to evaluate Gaussian distribution of values. According to the results, groups were analyzed by either parametric (data followed a Gaussian distribution) or non-parametric (data do not follow a Gaussian distribution) tests. Statistical differences between two groups were determined by Student’s t-test (parametric) or Mann-Whitney test (non-parametric). Statistical differences between more than two groups were determined by one-way ANOVA followed by multiple comparisons (parametric or non-parametric tests) *post-hoc* tests. The *p* value was considered significant ≤ 0.05. * *p* ≤ 0.05; ** *p* ≤ 0.01; *** *p* ≤ 0.001; **** *p* ≤ 0.0001; ns, not statistically significant. All experiments points are expressed as mean ± SEM. The number of samples (*N*), number of the replications of experiments and statistical analysis details (*P* and test used) are described in results section and legends of figures. The exclusion of values was performed by graphpad analysis of outlier’s tool. *In vivo* studies benefited from sample size analysis by the Gpower program. The treatment with paraquat was performed randomly between control (*Prkn*^*+/+*^) and *prkn* KO mice (*prkn*^−/−^).

## Supplementary Material

Supplementary figures R6 final1.docx

## Data and materials availability

All data are available in the main text or supplementary materials.

## References

[1] Cuervo AM, Stefanis L, Fredenburg R, et al. Impaired degradation of mutant alpha-synuclein by chaperone-mediated autophagy. Science. 2004;305(5688):1292–1295. doi: 10.1126/science.1101738 PubMed PMID: 15333840. 15333840

[2] Martinez-Vicente M, Talloczy Z, Kaushik S, et al. Dopamine-modified alpha-synuclein blocks chaperone-mediated autophagy. J Clin Invest. 2008;118(2):777–788. doi: 10.1172/JCI32806 PubMed PMID: 18172548; eng. 18172548 PMC2157565

[3] Vogiatzi T, Xilouri M, Vekrellis K, et al. Wild type alpha-synuclein is degraded by chaperone-mediated autophagy and macroautophagy in neuronal cells. J Biol Chem. 2008;283(35):23542–23556. doi: 10.1074/jbc.M801992200 PubMed PMID: 18566453; eng. 18566453 PMC2527094

[4] Oliveira LMA, Gasser T, Edwards R, et al. Alpha-synuclein research: defining strategic moves in the battle against Parkinson’s disease. NPJ Parkinsons Dis. 2021;7(1):65. doi: 10.1038/s41531-021-00203-9 PubMed PMID: 34312398. 34312398 PMC8313662

[5] Spillantini MG, Schmidt ML, Lee VM, et al. Alpha-synuclein in Lewy bodies. Nature. 1997;388(6645):839–840. PubMed PMID: 9278044. 9278044 10.1038/42166

[6] Sulzer D, Edwards RH. The physiological role of alpha-synuclein and its relationship to Parkinson’s disease. J Neurochem. 2019;150(5):475–486. doi: 10.1111/jnc.14810 PubMed PMID: 31269263; PubMed Central PMCID: PMCPMC6707892. 31269263 PMC6707892

[7] da Costa C, Ancolio K, Checler F. Wild-type but not Parkinson’s disease-related ala-53 –> thr mutant alpha -synuclein protects neuronal cells from apoptotic stimuli. J Biol Chem. 2000;275(31):24065–24069. doi: 10.1074/jbc.M002413200 10818098

[8] Surguchov A. Molecular and cellular biology of synucleins. Int Rev Cell Mol Biol. 2008;270:225–317. doi: 10.1016/S1937-6448(08)01406-8 PubMed PMID: 19081538; eng. 19081538

[9] Da Costa A, Paitel E, Vincent B, et al. Alpha-synuclein lowers p53-dependent apoptotic response of neuronal cells. Abolishment by 6-hydroxydopamine and implication for Parkinson’s disease. J Biol Chem. 2002;277(52):50980–50984. doi: 10.1074/jbc.M207825200 12397073

[10] Alves da Costa C. Recent advances on alpha-synuclein cell biology: functions and dysfunctions. Curr Mol Med. 2003;3(1):17–24. PubMed PMID: 12558071; eng. 12558071 10.2174/1566524033361690

[11] Kuo SH, Tasset I, Cheng MM, et al. Mutant glucocerebrosidase impairs alpha-synuclein degradation by blockade of chaperone-mediated autophagy. Sci Adv. 2022;8(6):eabm6393. doi: 10.1126/sciadv.abm6393 PubMed PMID: 35138901. 35138901 PMC11809618

[12] Kingsbury AE, Daniel SE, Sangha H, et al. Alteration in alpha-synuclein mRNA expression in Parkinson’s disease. Mov Disord. 2004;19(2):162–170. PubMed PMID: 14978671. 14978671 10.1002/mds.10683

[13] Neystat M, Lynch T, Przedborski S, et al. Alpha-synuclein expression in substantia nigra and cortex in Parkinson’s disease. Mov Disord. 1999;14(3):417–422. PubMed PMID: 10348463; eng. 10348463 10.1002/1531-8257(199905)14:3<417::aid-mds1005>3.0.co;2-x

[14] Keo A, Mahfouz A, Ingrassia AMT, et al. Transcriptomic signatures of brain regional vulnerability to Parkinson’s disease. Commun Biol. 2020;3(1):101. doi: 10.1038/s42003-020-0804-9 PubMed PMID: 32139796; PubMed Central PMCID: PMCPMC7058608. 32139796 PMC7058608

[15] Emin D, Zhang YP, Lobanova E, et al. Small soluble alpha-synuclein aggregates are the toxic species in Parkinson’s disease. Nat Commun. 2022;13(1):5512. doi: 10.1038/s41467-022-33252-6 PubMed PMID: 36127374; PubMed Central PMCID: PMCPMC9489799. 36127374 PMC9489799

[16] Shimura H, Hattori N, Kubo S, et al. Familial Parkinson disease gene product, parkin, is a ubiquitin-protein ligase. Nat Genet. 2000;25(3):302–305. doi: 10.1038/77060 PubMed PMID: 10888878. 10888878

[17] da Costa C, Sunyach C, Giaime E, et al. Transcriptional repression of p53 by parkin and impairment by mutations associated with autosomal recessive juvenile Parkinson’s disease. Nat Cell Biol. 2009;11(11):1370–1375. doi: 10.1038/ncb1981 19801972 PMC2952934

[18] Duplan E, Sevalle J, Viotti J, et al. Parkin differently regulates presenilin-1 and presenilin-2 functions by direct control of their promoter transcription. J Mol Cell Biol. 2013;5(2):132–142. doi: 10.1093/jmcb/mjt003 23359614

[19] Rouland L, Duplan E, Ramos Dos Santos L. Therapeutic potential of parkin as a tumor suppressor via transcriptional control of cyclins in glioblastoma cell and animal models. Theranostics. 2021;11(20):10047–10063. doi: 10.7150/thno.57549 PubMed PMID: 34815803; PubMed Central PMCID: PMCPMC8581414. 34815803 PMC8581414

[20] Ioghen OC, Ceafalan LC, Popescu BO. SH-SY5Y cell line in vitro models for Parkinson disease research-old practice for New trends. J Integr Neurosci. 2023;22(1):20. doi: 10.31083/j.jin2201020 PubMed PMID: 36722247. 36722247

[21] Mazzulli JR, Xu YH, Sun Y, et al. Gaucher disease glucocerebrosidase and alpha-synuclein form a bidirectional pathogenic loop in synucleinopathies. Cell. 2011;146(1):37–52. doi: 10.1016/j.cell.2011.06.001 PubMed PMID: 21700325; eng. 21700325 PMC3132082

[22] Kaushik S, Cuervo AM. The coming of age of chaperone-mediated autophagy. Nat Rev Mol Cell Biol. 2018;19(6):365–381. doi: 10.1038/s41580-018-0001-6 29626215 PMC6399518

[23] Kaushik S, Cuervo AM. Methods to monitor chaperone-mediated autophagy. Methods Enzymol. 2009;452:297–324. doi: 10.1016/S0076-6879(08)03619-7 PubMed PMID: 19200890; PubMed Central PMCID: PMCPMC4300957. 19200890 PMC4300957

[24] Vellingiri B, Chandrasekhar M, Sri Sabari S, et al. Neurotoxicity of pesticides - a link to neurodegeneration. Ecotoxicol Environ Saf. 2022;243:113972. doi: 10.1016/j.ecoenv.2022.113972 PubMed PMID: 36029574. 36029574

[25] Sunico CR, Nakamura T, Rockenstein E, et al. S-Nitrosylation of parkin as a novel regulator of p53-mediated neuronal cell death in sporadic Parkinson’s disease. Mol Neurodegener. 2013;8(1):29. doi: 10.1186/1750-1326-8-29 PubMed PMID: 23985028; PubMed Central PMCID: PMCPMC3765907. 23985028 PMC3765907

[26] Gandelman M, Dansithong W, Kales SC, et al. The AKT modulator A-443654 reduces alpha-synuclein expression and normalizes er stress and autophagy. J Biol Chem. 2021;297(4):101191. doi: 10.1016/j.jbc.2021.101191 PubMed PMID: 34520759; PubMed Central PMCID: PMCPMC8482485. 34520759 PMC8482485

[27] Spillantini MG, Crowther RA, Jakes R, et al. A-synuclein in filamentous inclusions of Lewy bodies from Parkinson’s disease and dementia with Lewy bodies. Proc Natl Acad Sci USA. 1998;95(11):6469–6473. doi: 10.1073/pnas.95.11.6469 9600990 PMC27806

[28] Hijaz BA, Volpicelli-Daley LA. Initiation and propagation of alpha-synuclein aggregation in the nervous system. Mol Neurodegener. 2020;15(1):19. doi: 10.1186/s13024-020-00368-6 PubMed PMID: 32143659; PubMed Central PMCID: PMCPMC7060612. 32143659 PMC7060612

[29] Bras IC, Outeiro TF. Alpha-Synuclein: mechanisms of release and pathology progression in Synucleinopathies. Cells. 2021;10(2). doi: 10.3390/cells10020375 PubMed PMID: 33673034; PubMed Central PMCID: PMCPMC7917664. PMC7917664 33673034

[30] Zondler L, Kostka M, Garidel P, et al. Proteasome impairment by alpha-synuclein. PLOS ONE. 2017;12(9):e0184040. doi: 10.1371/journal.pone.0184040 PubMed PMID: 28945746; PubMed Central PMCID: PMCPMC5612461. 28945746 PMC5612461

[31] Fujiwara H, Hasegawa M, Dohmae N, et al. A-synuclein is phosphorylated in synucleinopathy lesions. Nat Cell Biol. 2002;4(2):160–164. doi: 10.1038/ncb748 11813001

[32] Chung KK, Zhang Y, Lim KL, et al. Parkin ubiquitinates the alpha-synuclein-interacting protein, synphilin-1: implications for Lewy-body formation in Parkinson disease. Nat Med. 2001;7(10):1144–1150. doi: 10.1038/nm1001-1144 PubMed PMID: 11590439. 11590439

[33] Khandelwal PJ, Dumanis SB, Feng LR, et al. Parkinson-related parkin reduces alpha-synuclein phosphorylation in a gene transfer model. Mol Neurodegener. 2010;5(1):47. doi: 10.1186/1750-1326-5-47 PubMed PMID: 21050448; PubMed Central PMCID: PMCPMC2987994. 21050448 PMC2987994

[34] Jiang H, Jiang Q, Liu W, et al. Parkin suppresses the expression of monoamine oxidases. J Biol Chem. 2006;281(13):8591–8599. doi: 10.1074/jbc.M510926200 PubMed PMID: 16455660. 16455660

[35] Jiang H, Ren Y, Yuen E, et al. Parkin controls dopamine utilization in human midbrain dopaminergic neurons derived from induced pluripotent stem cells. Nat Commun. 2012;3(1):668. doi: 10.1038/ncomms1669 22314364 PMC3498452

[36] Madsen DA, Schmidt SI, Blaabjerg M, et al. Interaction between Parkin and alpha-synuclein in PARK2-mediated Parkinson’s disease. Cells. 2021;10(2):283. doi: 10.3390/cells10020283 PubMed PMID: 33572534; PubMed Central PMCID: PMCPMC7911026. 33572534 PMC7911026

[37] Jesko H, Lenkiewicz AM, Wilkaniec A, et al. The interplay between parkin and alpha-synuclein; possible implications for the pathogenesis of Parkinson’s disease. Acta Neurobiol Exp (Wars). 2019;79(3):276–289. PubMed PMID: 31587020. 31587020

[38] Sidransky E, Lopez G. The link between the GBA gene and parkinsonism. Lancet Neurol. 2012;11(11):986–998. doi: 10.1016/S1474-4422(12)70190-4 PubMed PMID: 23079555; eng. 23079555 PMC4141416

[39] Hoglinger G, Schulte C, Jost WH, et al. GBA-associated pd: chances and obstacles for targeted treatment strategies. J Neural Transm (Vienna). 2022;129(9):1219–1233. doi: 10.1007/s00702-022-02511-7 PubMed PMID: 35639160; PubMed Central PMCID: PMCPMC9463270. 35639160 PMC9463270

[40] Miyoshi K, Hagita H, Horiguchi T, et al. Redefining GBA gene structure unveils the ability of cap-independent, IRES-dependent gene regulation. Commun Biol. 2022;5(1):639. doi: 10.1038/s42003-022-03577-5 PubMed PMID: 35831491; PubMed Central PMCID: PMCPMC9279297. 35831491 PMC9279297

[41] Horowitz M, Wilder S, Horowitz Z, et al. The human glucocerebrosidase gene and pseudogene: structure and evolution. Genomics. 1989;4(1):87–96. doi: 10.1016/0888-7543(89)90319-4 PubMed PMID: 2914709. 2914709

[42] Kamienieva I, Duszynski J, Szczepanowska J. Multitasking guardian of mitochondrial quality: Parkin function and Parkinson’s disease. Transl Neurodegener. 2021;10(1):5. doi: 10.1186/s40035-020-00229-8 PubMed PMID: 33468256; PubMed Central PMCID: PMCPMC7816312. 33468256 PMC7816312

[43] Sardiello M, Palmieri M, di Ronza A, et al. A gene network regulating lysosomal biogenesis and function. Science. 2009;325(5939):473–477. doi: 10.1126/science.1174447 PubMed PMID: 19556463. 19556463

[44] Wang K, Zhang B, Zhang B, et al. Paraquat inhibits autophagy via intensifying the interaction between HMGB1 and alpha-synuclein. Neurotox Res. 2022;40(2):520–529. doi: 10.1007/s12640-022-00490-x PubMed PMID: 35316522. 35316522

[45] Nakamura T, Oh CK, Zhang X, et al. Protein S-nitrosylation and oxidation contribute to protein misfolding in neurodegeneration. Free Radic Biol Med. 2021;172:562–577. doi: 10.1016/j.freeradbiomed.2021.07.002 PubMed PMID: 34224817; PubMed Central PMCID: PMCPMC8579830. 34224817 PMC8579830

[46] Aquilano K, Baldelli S, Rotilio G, et al. Role of nitric oxide synthases in Parkinson’s disease: a review on the antioxidant and anti-inflammatory activity of polyphenols. Neurochem Res. 2008;33(12):2416–2426. doi: 10.1007/s11064-008-9697-6 PubMed PMID: 18415676. 18415676

[47] Sircar E, Rai SR, Wilson MA, et al. Neurodegeneration: impact of S-nitrosylated parkin, DJ-1 and PINK1 on the pathogenesis of Parkinson’s disease. Arch Biochem Biophys. 2021;704:108869. doi: 10.1016/j.abb.2021.108869 PubMed PMID: 33819447. 33819447

[48] Wilkaniec A, Lenkiewicz AM, Czapski GA, et al. Extracellular alpha-synuclein oligomers induce parkin S-Nitrosylation: relevance to sporadic Parkinson’s disease etiopathology. Mol Neurobiol. 2019;56(1):125–140. doi: 10.1007/s12035-018-1082-0 PubMed PMID: 29681024; PubMed Central PMCID: PMCPMC6334739. 29681024 PMC6334739

[49] Duplan E, Giaime E, Viotti J, et al. ER-stress-associated functional link between Parkin and DJ-1 via a transcriptional cascade involving the tumor suppressor p53 and the spliced X-box binding protein XBP-1. J Cell Sci. 2013;126(Pt 9):2124–2133. doi: 10.1242/jcs.127340 23447676 PMC6518232

[50] Duplan E, Giordano C, Checler F, et al. Direct alpha-synuclein promoter transactivation by the tumor suppressor p53. Mol Neurodegener. 2016;11(1):13. doi: 10.1186/s13024-016-0079-2 PubMed PMID: 26833254; PubMed Central PMCID: PMCPMC4736712. 26833254 PMC4736712

[51] Mates L, Chuah MK, Belay E, et al. Molecular evolution of a novel hyperactive sleeping beauty transposase enables robust stable gene transfer in vertebrates. Nat Genet. 2009;41(6):753–761. doi: 10.1038/ng.343 PubMed PMID: 19412179. 19412179

[52] Itier JM, Ibanez P, Mena MA, et al. Parkin gene inactivation alters behaviour and dopamine neurotransmission in the mouse. Hum Mol Genet. 2003;12(18):2277–2291. doi: 10.1093/hmg/ddg239 PubMed PMID: 12915482. 12915482

[53] Xu J. Preparation, culture, and immortalization of mouse embryonic fibroblasts. Curr Protoc Mol Biol. 2005;70(1). doi: 10.1002/0471142727.mb2801s70 PubMed PMID: 18265366. 18265366

[54] Gautier CA, Erpapazoglou Z, Mouton-Liger F, et al. The endoplasmic reticulum-mitochondria interface is perturbed in PARK2 knockout mice and patients with PARK2 mutations. Hum Mol Genet. 2016;25(14):ddw148. doi: 10.1093/hmg/ddw148 27206984

[55] Kim S, Yun SP, Lee S, et al. GBA1 deficiency negatively affects physiological alpha-synuclein tetramers and related multimers. Proc Natl Acad Sci USA. 2018;115(4):798–803. doi: 10.1073/pnas.1700465115 PubMed PMID: 29311330; PubMed Central PMCID: PMCPMC5789900. 29311330 PMC5789900

[56] Lazaro DF, Rodrigues EF, Langohr R, et al. Systematic comparison of the effects of alpha-synuclein mutations on its oligomerization and aggregation. PLOS Genet. 2014;10(11):e1004741. doi: 10.1371/journal.pgen.1004741 PubMed PMID: WOS:000345455200011. 25393002 PMC4230739

[57] Allan C, Burel JM, Moore J, et al. OMERO: flexible, model-driven data management for experimental biology. Nat Methods. 2012;9(3):245–253. doi: 10.1038/nmeth.1896 PubMed PMID: 22373911; PubMed Central PMCID: PMCPMC3437820. 22373911 PMC3437820

[58] Bolte S, Cordelieres FP. A guided tour into subcellular colocalization analysis in light microscopy. J Microsc. 2006;224(Pt 3):213–232. doi: 10.1111/j.1365-2818.2006.01706.x PubMed PMID: 17210054. 17210054

[59] Schneider CA, Rasband WS, Eliceiri KW. NIH image to ImageJ: 25 years of image analysis. Nat Methods. 2012;9(7):671–675. doi: 10.1038/nmeth.2089 PubMed PMID: 22930834; PubMed Central PMCID: PMCPMC5554542. 22930834 PMC5554542

[60] Pouchin P, Zoghlami R, Valarcher R, et al. Easing batch image processing from OMERO: a new toolbox for ImageJ. F1000Res. 2022;11:392. doi: 10.12688/f1000research.110385.1 PubMed PMID: 35685190; PubMed Central PMCID: PMCPMC9171289. 35685190 PMC9171289

