# Supplementary material for: PRKN/parkin-mediated control of SNCA (synuclein alpha) and chaperone-mediated autophagy are defective in cellular, mice models and Parkinson disease-affected brains
Source: Autophagy. 2026 May 13;22(9):2405–23. doi: 10.1080/15548627.2026.2669458 (PMC13502014; doi:10.1080/15548627.2026.2669458)
Supplement: Supplementary file 1 [file KAUP_A_2669458_SM9074.docx]

**Supplementary Figures**

**Figure S1.** Endogenous PRKN regulates SNCA in neuroblastoma cells. SH-SY5Y cells stably expressing scramble (*PRKN [SC])* or *PRKN*-directed shRNA (*PRKN [KD])* were examined for PRKN (**A and B**) and SNCA [M] (**A and C**) protein expressions and *SNCA* mRNA levels (**D**). Bars are expressed as percent of control *PRKN* [SC] cells (taken as 100) and are the means ± SEM. (**E**) Human and murine *SNCA/Snca* promoter regions were used to assess promoter activity in *PRKN [SC]* and *PRKN [KD]* cells. **(F)** Wild-type (*WTPr*) and *PRKN*-RE-deleted (Δ*PkPr***)** *SNCA* promoter activities measured in *PRKN [SC]* and *PRKN [KD].* In **E and F** bars are expressed as percent of control *PRKN [SC]* cells (taken as 100) and are the means ± SEM. ***P* ≤ 0.01; ****P* ≤ 0.001; **** *P* ≤ 0.0001; ns: not statistically significant. All full gels are presented in Data S7.

**Figure S2.** PRKN does not physically interact with Site 2 and 3 *PRKN*-RE of *SNCA* promoter. ChIP analysis of the physical interaction between endogenous SNCA site 2 (**A**) and site 3 (**B**) and PRKN in PRKN-overexpressing SH-SY5Y cells assessed by means of control (IgG, black bars) or PRKN-directed antibodies (anti-PRKN, gray bars) as described in Methods. All bars represent the means ± SEM.

**Figure S3.** GBA1 is regulated by endogenous PRKN**.** Fibroblasts derived from *Prkn^+/+^* and *prkn^-/-^* mice were assessed for GBA1 protein (**A** and **B)** and activity (**C**). Bars are expressed as percent of control *Prkn^+/+^* cells taken as 100 and are the means ± SEM. Protein loading was examined by stain free membranes (SFM). (**D**) Quantification of *Gba1* mRNA levels. Bars are expressed as percent of control *prkn*^-/-^ cells (taken as 100) and are the means ± SEM. *Gba1* promoter activity carried out with *P1* and *P2* promoter vectors bearing similar (*PRKN*-RE) sites (**E**) or with *P2* deleted of the *PRKN*-RE (5’-CTCCGGC-3’, P2Δ) (**F**). Bars are expressed as percent of control *Prkn*^+/+^ cells (taken as 100) and are the means ± SEM. **P* ≤ 0.05; ****P* ≤ 0.001; *****P* ≤ 0.0001; ns: not statistically significant. All full gels are presented in Data S8.

**Figure S4.** CMA activity is controlled by endogenous PRKN in human neuroblastoma. *PRKN [SC]* and *PRKN [KD]* SH-SY5Y cells were assessed for LAMP2A and HSPA8 (**A and B**) protein expressions and *LAMP2A* mRNA (**C**). Protein loading is illustrated by Stain free membrane (SFM). Bars are expressed as percent of control *PRKN [SC]* cells (taken as 100) and are the means ± SEM. (**D)** Histological analysis of LAMP2 and HSPA8 colocalization with anti-LAMP2 (green) and HSPA8 (red) and DAPI (nuclei, blue). Merged images of all tree channels are indicated and ZOOM correspond to the squared zones indicated in merge images. Scale bars: 10 μm. **E,** quantification of LAMP2/HSPA8 colocalization in lysosomes analyzed by Pearson's and Manders correlation coefficients. **P* ≤ 0.05; ***P* ≤ 0.01; ****P* ≤ 0.001; *****P* ≤ 0.0001; ns: not statistically significant. All full gels are presented in Data S9.

**Figure S5.** Direct and indirect GBA-mediated control of SNCA and CMA by PRKN. Scheme created with Biorender.

**Data S1.** Full gels Figure 1.

**Data S2.** Full gels Figure 3.

**Data S3.** Full gels Figure 4.

**Data S4.** Full gels Figure 5.

**Data S5.** Full gels Figure 6.

**Data S6.** Full gels Figure 7.

**Data S7.** Full gels Figure S1.

**Data S8.** Full gels Figure S3.

**Data S9.** Full gels Figure S4.
